# Expanding the Phase Space for Halide-Based Solid Electrolytes: Li–Mg–Zr–Cl Spinels

**DOI:** 10.1021/acs.chemmater.4c01160

**Published:** 2024-07-26

**Authors:** Christopher L. Rom, Philip Yox, Abby M. Cardoza, Rebecca W. Smaha, Maxwell Q. Phan, Trevor R. Martin, Annalise E. Maughan

**Affiliations:** † National Renewable Energy Laboratory, Golden, Colorado 80401, United States; ‡ Department of Chemistry, Colorado School of Mines, Golden, Colorado 80401, United States

## Abstract

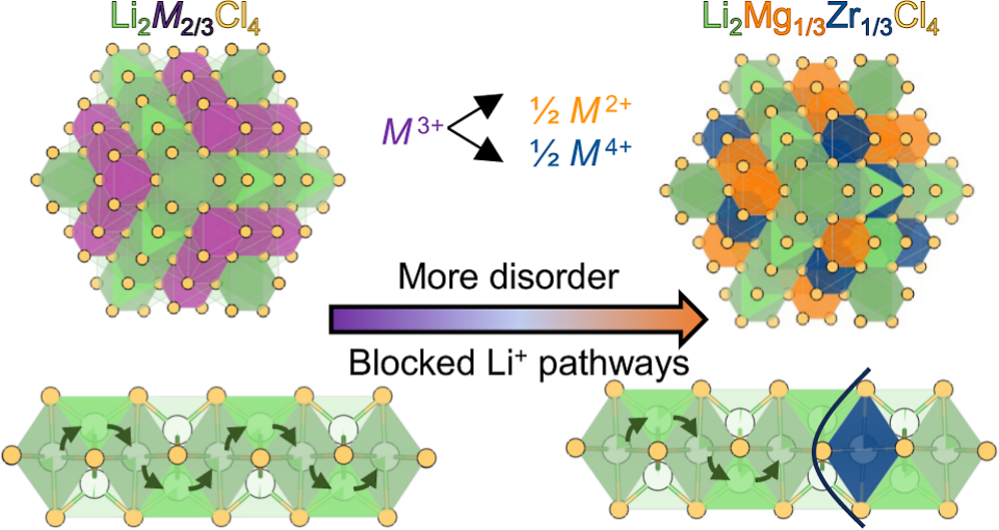

Chloride-based solid electrolytes are intriguing materials owing to their high Li^+^ ionic conductivity and electrochemical compatibility with high-voltage oxide cathodes for all-solid-state lithium batteries. However, the leading examples of these materials are limited to trivalent metals (e.g., Sc, Y, and In), which are expensive and scarce. Here, we expand this materials family by replacing the trivalent metals with a mix of di- and tetra-valent metals (e.g., Mg^2+^ and Zr^4+^). We synthesize Li_2_Mg_1/3_Zr_1/3_Cl_4_ in the spinel crystal structure and compare its properties with the high-performing Li_2_Sc_2/3_Cl_4_ that has been reported previously. We find that Li_2_Mg_1/3_Zr_1/3_Cl_4_ has lower ionic conductivity (0.028 mS/cm at 30 °C) than the isostructural Li_2_Sc_2/3_Cl_4_ (1.6 mS/cm at 30 °C). We attribute this difference to a disordered arrangement of Mg^2+^ and Zr^4+^ in Li_2_Mg_1/3_Zr_1/3_Cl_4_, which may block Li^+^ migration pathways. However, we show that aliovalent substitution across the Li_2–*z*_Mg_1–3*z*/2_Zr_*z*_Cl_4_ series between Li_2_MgCl_4_ and Li_2_ZrCl_6_ can boost ionic conductivity with increasing Zr^4+^ content, presumably due to the introduction of Li^+^ vacancies. This work opens a new dimension for halide-based solid electrolytes, accelerating the development of low-cost solid-state batteries.

## Introduction

Solid electrolytes are the key material needed to enable the production of all-solid-state batteries. Replacing the flammable organic liquids currently used as electrolytes in lithium-ion batteries with a nonflammable inorganic solid can mitigate fire hazards and thermal runaway.^1^ Additionally, using solid electrolytes may enable the use lithium-metal anodes and dramatically increase battery energy density.^1,2^ Therefore, the development of low-cost, high-performance solid electrolytes is essential for realizing these potential advances in battery technology.

Chloride-based solid electrolytes are a particularly interesting class of solid electrolytes, as many exhibit high ionic conductivities and large voltage stability windows.^3,4^ Since the 2018 report of high ionic conductivity of 0.51 mS/cm in Li_3_YCl_6_,^5^ numerous chlorides have been reported with ionic conductivities exceeding 1 mS/cm (e.g., Li_3_ScCl_6_,^6^ Li_2_Sc_2/3_Cl_4_,^7^ Li_3_InCl_6_^8^). When using fully oxidized cations (e.g., Zr^4+^), the oxidative stability window of chlorides is limited by the oxidation of the chloride anion (2Cl^–^ → Cl_2_ + 2e^–^) at approximately 4.3 V versus Li/Li^+^.^9^ This window is far wider than that of sulfide or bromide electrolytes, and safely encompasses the range of high-voltage oxide cathodes.^9,10^ Thus, chlorides may be stable in contact with uncoated cathode materials without undergoing detrimental side reactions. However, the high-performing materials reported so far often rely on trivalent metals (e.g., Sc, Y, In, rare earth elements),^3^ which are expensive and scarce.^11^

Expanding this material space may be possible by substituting trivalent metals for even mixtures of di- and tetra-valent cations (i.e., replacing M^3+^ with ). We take inspiration from semiconductor research, where the high-performing III–V materials class (e.g, GaN) has been successfully extended via a highly tunable class of II–IV–V_2_ materials (e.g., MgSnN_2_).^12,13^ One advantage of this approach is that expensive elements may be replaced with more economically viable alternatives. The high-performing Sc-based electrolytes (e.g., Li_3_ScCl_6_ and Li_2_Sc_2/3_Cl_4_)^6,7^ are a prime target for this strategy, as Sc is produced in small quantities globally and typically demands a high price (>$200/g for ScCl_3_, Table S1). Despite a crustal abundance higher than Pb, Sc tends not to concentrate in ores and is therefore difficult to mine at scale.^14^ The high ionic conductivity of the inverse spinel Li_2_Sc_2/3_Cl_4_ and the scarcity and cost of Sc motivated our search for alternative spinel chemistries with similar performance metrics.

Here, we report a new family of halospinel electrolytes: Li_2_Mg_1/3_Zr_1/3_Cl_4_ and Li_2–*z*_Mg_1–3*z*/2_Zr_*z*_Cl_4_ (0 < *z* < 2/3). Synchrotron powder X-ray diffraction (SPXRD) reveals that ball-milling binary chlorides produces quaternary compounds with the inverse spinel crystal structure for compositions of Li_2_Mg_1/3_Zr_1/3_Cl_4_ and the solid-solution series of Li_2–*z*_Mg_1–3*z*/2_Zr_*z*_Cl_4_ up to *z* = 0.4 (i.e., Li_1.4_Mg_0.4_Zr_0.4_Cl_4_). For target compositions above *z* = 0.4, we observe phase separation into the inverse spinel phase and the heterostructural Li_2_ZrCl_6_ that indicates a solubility limit of Zr^4+^ into the inverse spinel structure. Electrochemical impedance spectroscopy (EIS) shows that the Li_2_Mg_1/3_Zr_1/3_Cl_4_ spinel exhibits lower ionic conductivity than the Li_2_Sc_2/3_Cl_4_ spinel that was previously reported (σ_i,30 °C_ = 0.028 and 1.6 mS/cm, respectively). Our SPXRD measurements suggest that cation disorder in the Li_2_Mg_1/3_Zr_1/3_Cl_4_ spinel may be responsible for the lower ionic conductivity. However, aliovalent substitution of Zr^4+^ into Li_2_MgCl_4_ can tune ionic conductivity of the Li_2–*z*_Mg_1–3*z*/2_Zr_*z*_Cl_4_ series across 3 orders of magnitude (i.e., from 10^–4^ to >10^–1^ mS/cm), with a maximum observed value of 0.43 mS/cm at *z* = 0.57. While these ionic conductivity values are below the generally accepted 1 mS/cm criterion for practical solid-state electrolytes, these findings point to a new area of research into halide-based solid-ion conductors focused on II–IV substitution for the III metals commonly used to date.

## Results and Discussion

### Mechanochemical Synthesis and Structure of Li–Mg–Zr–Cl Phases

We prepared a new solid electrolyte Li_2_Mg_1/3_Zr_1/3_Cl_4_ through high-energy planetary ball-milling. Li_2_Mg_1/3_Zr_1/3_Cl_4_ adopts the inverse spinel structure similar to Li_2_MgCl_4_ (space group *Fd*3̅*mZ*, Figure 2).^15^ In the archetypal inverse halospinel Li_2_MgCl_4_, Mg^2+^ cations share the 16d octahedral site with Li^+^ cations and additional Li^+^ cations reside on the tetrahedral 8a site, as shown in Figure 2a. Using the Li_2_MgCl_4_ model (ICSD #74957)^15^ as a framework, we constructed an initial structural model of Li_2_Mg_1/3_Zr_1/3_Cl_4_ by introducing Zr^4+^ cations onto the 16d site. To reflect the input stoichiometry, the occupancies of the Mg^2+^ and Zr^4+^ on the 16d site were initially set to 1/6 (i.e., 0.1667).

**Figure 1:**
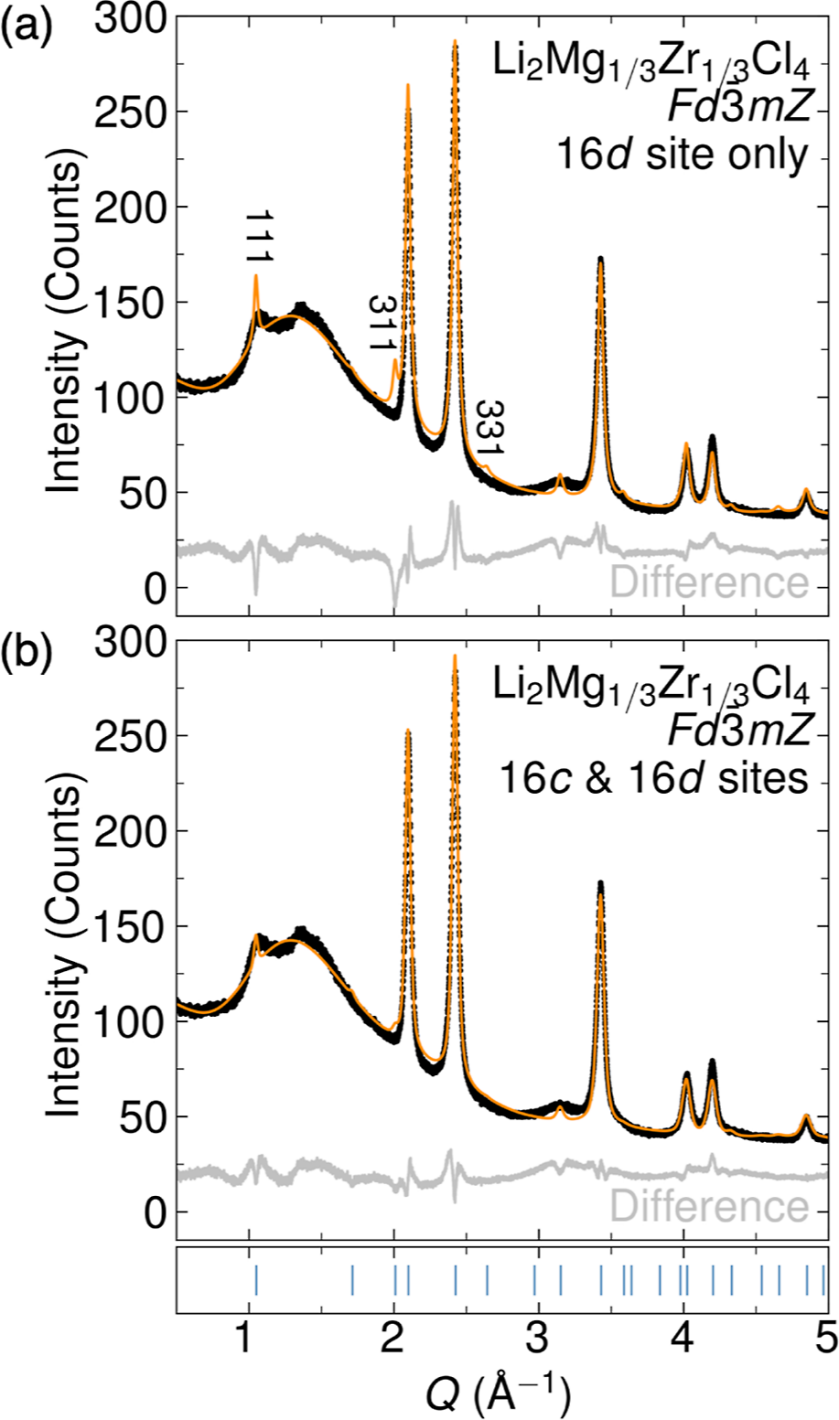
SPXRD pattern of Li_2_Mg_1/3_Zr_1/3_Cl_4_ prepared by high-energy ball-milling, contrasted with two structural models: Rietveld refinement of the data to an inverse halospinel structure (a) in which Mg^2+^ and Zr^4+^ reside only on the 16d sites, which results in poor fitting of the (111), (311), and (331) reflections (*R*_wp_ = 5.10%), contrasted with (b) a structure with Mg^2+^ and Zr^4+^ disordered on both the 16d and 16c sites (*R*_wp_ = 3.89%). Corresponding structural models are shown in Figure 2. In (a,b), the black circles are the data, the fit is the orange line, and the difference curve is shown in gray. The positions of anticipated reflections for the spinel structure are shown as blue ticks in the subpanel. Figure S2 shows the SPXRD data with the full *Q* range.

**Figure 2:**
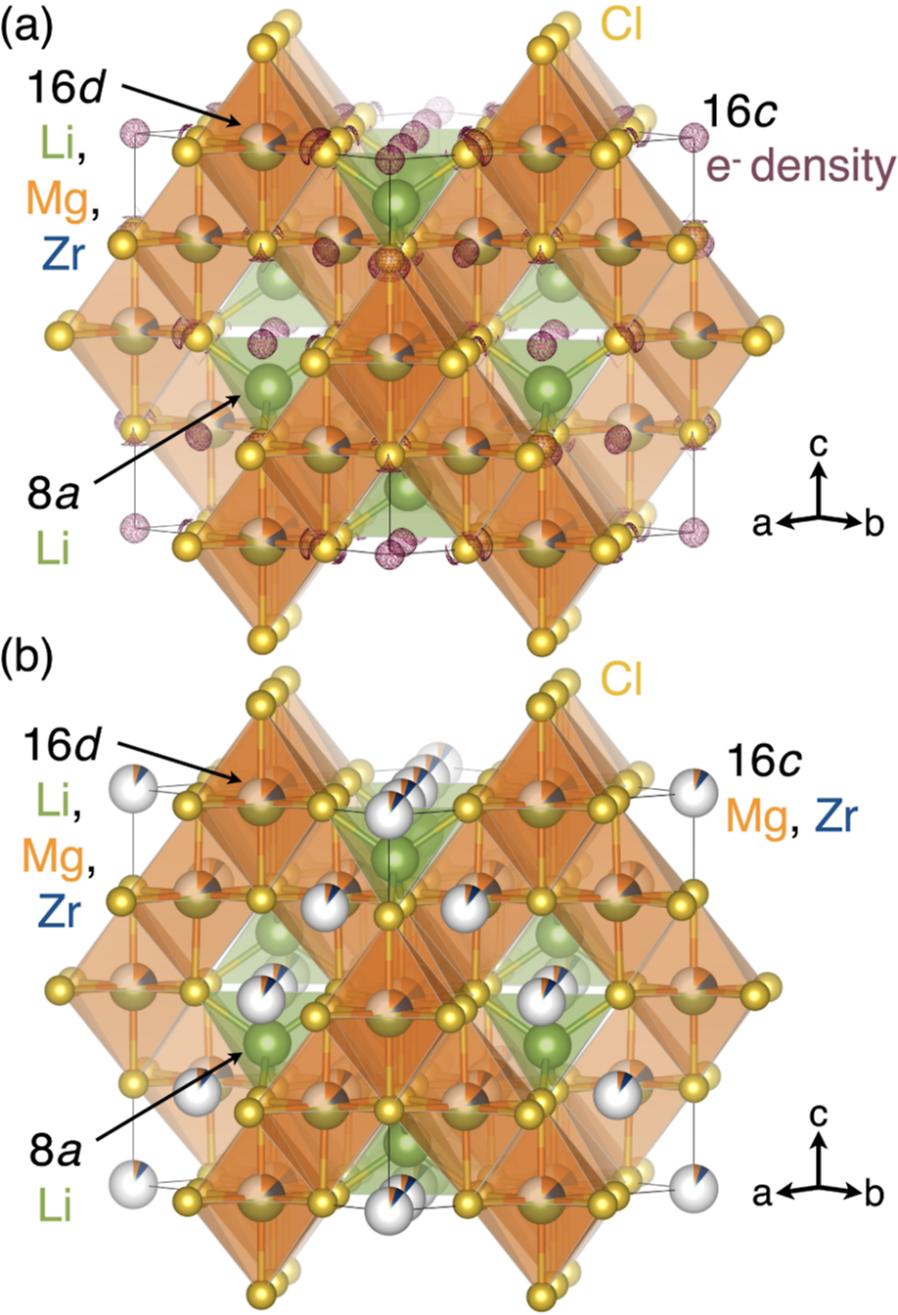
Structural models of the halospinel Li_2_Mg_1/3_Zr_1/3_Cl_4_ generated from Rietveld refinements shown in Figure 1. In (a), a positive Fourier electron density difference map (shown as magenta wireframe) is superposed on the spinel structural model and reveals residual electron density on the 16c site. In (b), incorporation of Mg^2+^ and Zr^4+^ onto the 16c site of the spinel structure improves the fit to the SPXRD data (Figure 1b).

SPXRD data of Li_2_Mg_1/3_Zr_1/3_Cl_4_ are reasonably well-described by the spinel structure (Figures 1a and 2a). The low peak intensity and wide peak breadth are suggestive of small crystalline domain lengths, as is commonly observed in materials prepared by mechanochemical synthesis.^16,17^ The broad feature between *Q* = 1.0 and 2.0 Å^–1^ is attributed to the quartz capillary. From initial Rietveld refinement (Figure 1a), we find that Li_2_Mg_1/3_Zr_1/3_Cl_4_ adopts the inverse spinel structure with cubic lattice parameter *a* = 10.3706(3) Å, which is smaller than the reported lattice parameters for Li_2_MgCl_4_ (*a* = 10.401(2) Å)^15^ and Li_2_Sc_2/3_Cl_4_ (*a* = 10.4037(5) Å).^7^ The fractional occupancies of the Mg^2+^ and Zr^4+^ cations on the 16d sites were constrained to be equivalent and refined to a value of 0.151(1). This refined occupancy is slightly reduced relative to the initial value of 0.1667 (i.e., 1/6) determined by the input stoichiometry of Li_2_Mg_1/3_Zr_1/3_Cl_4_ and may reflect a degree of cation site disorder or the presence of an amorphous phase. Li^+^ occupancies and atomic displacement parameters were not refined due to the low X-ray scattering cross section of Li^+^. The resultant Rietveld refinement and refined structural model are shown in Figures 1a and 2a, respectively, and the refined parameters are in Table 1.

**Table 1:** Results of the Rietveld Refinement to SPXRD Data of Li_2_Mg_1/3_Zr_1/3_Cl_4_ Shown in Figure 1a^a^

site	atom	*x*	*y*	*z*	occ	*U*_11=22=33_ (Å^2^)	*U*_12=13=23_ (Å^2^)
8a	Li	0.125	0.125	0.125	1	0.04	
16d	Li	0.5	0.5	0.5	0.5	0.04	
16d	Mg	0.5	0.5	0.5	0.151(1)	0.023(2)	0.024(7)
16d	Zr	0.5	0.5	0.5	0.151(1)	0.023(2)	0.024(7)
32e	Cl	0.2518(1)	0.2518(1)	0.2518(1)	1	0.0251(3)	0.001(5)

a Space group *Fd*3̅*mZ*, *a* = 10.3706(3) Å. *R*_wp_ = 5.10%.

Interestingly, we find that the intensities of several reflections—namely the (111), (311), and (331)—are overestimated in the initial refined model relative to the data (Figure 1a). In order to determine the origin of this observation, we generated a Fourier difference map to visualize unaccounted-for electron density in the structure. The positive Fourier difference map is shown as the magenta isosurface superposed on the refined structural model in Figure 2a and reveals additional electron density at the (0,0,0) position (16c site) within the spinel structure. Given the reduced occupancies of Mg^2+^ and Zr^4+^ from the initial Rietveld refinement, we suspect that this residual electron density is due to occupation of Mg^2+^ and Zr^4+^ on the 16c octahedral site. Cation disorder across additional sites is not uncommon in the spinel structure. In the halospinel Li_2_Sc_2/3_Cl_4_, the Li^+^ cations partially occupy two additional sites in the spinel structure—a second tetrahedral site (48f) and a second octahedral 16c site—as determined by neutron powder diffraction.^7^ Site disorder between the 16d and 16c octahedral sites has also been previously observed in spinel oxides used as battery cathodes.^18,19^

In order to determine the degree of cation site mixing between the 16d and 16c sites, we performed a Rietveld refinement of the Li_2_Mg_1/3_Zr_1/3_Cl_4_ spinel structure with Mg^2+^ and Zr^4+^ occupying both the 16d and 16c sites. The fractional occupancies between the cations across both sites were constrained to maintain the input stoichiometry Li_2_Mg_1/3_Zr_1/3_Cl_4_. Introduction of Mg^2+^/Zr^4+^ on the 16c site substantially improves the fit to the SPXRD data (from *R*_wp_ = 5.10 to 3.89%), as shown in the Rietveld refinement in Figure 1b. The refined model is shown in Figure 2b with Mg^2+^ occupancies of 16d = 0.12(23) and 16c = 0.05(23) and Zr^4+^ occupancies of 16d = 0.11(7) and 16c = 0.06(7). Refined parameters for the structural model with partial Mg^2+^/Zr^4+^ on the 16c site are shown in Table 2. We also performed an unconstrained refinement of Mg^2+^ and Zr^4+^ on the 16d site only (Figure S3, Table S2), which did not improve the fit quality compared to the analysis presented in Figure 1b and Table 2.

**Table 2:** Results of the Rietveld Refinement to SPXRD Data of Li_2_Mg_1/3_Zr_1/3_Cl_4_ Shown in Figure 1b, with Mg and Zr Allowed to Refine on the 16c and 16d Sites^a^

site	atom	*x*	*y*	*z*	occ	*U*_11=22=33_ (Å^2^)	*U*_12=13=23_ (Å^2^)
8a	Li	0.125	0.125	0.125	1	0.04	
16d	Li	0.5	0.5	0.5	0.5	0.04	
16d	Mg	0.5	0.5	0.5	0.12(23)	0.030(4)	0.029(9)
16d	Zr	0.5	0.5	0.5	0.11(7)	0.030(4)	0.029(9)
16c	Mg	0	0	0	0.05(23)	0.007(5)	
16c	Zr	0	0	0	0.06(7)	0.007(5)	
32e	Cl	0.2547(2)	0.2547(2)	0.2547(2)	1	0.0235(3)	0.006(3)

a Space group *Fd*3̅*mZ*, *a* = 10.3717(3) Å. *R*_wp_ = 3.89%. The CIF is included in the Supporting Information.

Keen readers will note that the uncertainty values in the occupancies for both cations are larger than the refined values; in fact, the 16c occupancy values are within error of 0. However, this does not mean the 16c site is empty, within error. Rather, the high uncertainty is an artifact of the covariance between Mg^2+^ and Zr^4+^ occupancy values. To visualize this, we performed parametric refinements with Mg^2+^ and Zr^4+^ occupancy systematically varied across the 16d and 16c sites (Figure S4). This analysis shows that a substantial range of Mg^2+^ and Zr^4+^ occupancy values provide similar quality fits, so long as some electron-density is present on the 16c site (roughly equivalent to 1/6 of a Mg^2+^ ion, or approximately 2 electrons). Additionally, Rietveld analysis of ball-milled Li_2_MgCl_4_ (without the problem of Mg^2+^/Zr^4+^ covariance) shows much lower uncertainty values for the Mg^2+^ 16d and 16c site occupancies: 0.392(2) and 0.108(2), respectively (Figure S6, see CIFs in Supporting Information). Furthermore, an amorphous secondary phase is unlikely to account for other Mg^2+^ or Zr^4+^ content, as quantitative Rietveld analysis suggests that the spinel phase accounts for all of the sample (Figure S10). However, neutron diffraction measurements will be essential for the full characterization of this new material, particularly as X-rays are insensitive to Li occupancy. Nevertheless, the observation of residual electron density in the Fourier difference map combined with the improvement in the fit support the notion of cation occupation on the additional 16c site in the spinel Li_2_Mg_1/3_Zr_1/3_Cl_4_.

The difference in octahedral occupancies between Li_2_Mg_1/3_Zr_1/3_Cl_4_ (16d and 16c disorder) compared to Li_2_MgCl_4_ and Li_2_Sc_2/3_Cl_4_ may stem from the synthetic methods. Li_2_Sc_2/3_Cl_4_ and Li_2_MgCl_4_ were synthesized via slow cooling from a fully molten phase at ca. 600 °C.^7,15^ This slow cooling likely allowed the most highly charged cations (Sc^3+^ and Mg^2+^, respectively) sufficient time to diffuse into their energetically preferred site (16d). In contrast, mechanochemical synthesis is well-known to induce cation disorder in other ternary metal chlorides such as Li_3_YCl_6_ and Li_3_ErCl_6_, which can then be tuned via annealing.^16^ Although gentle heating of Li_2_Mg_1/3_Zr_1/3_Cl_4_ drives phase separation due to the high volatility of ZrCl_4_ (Figure S5), ball-milled Li_2_MgCl_4_ (with ca. 20% of the Mg^2+^ disordered on the 16c site) was annealed at 260 °C for 12 h without decomposition. The annealed phase exhibited much sharper Bragg peaks, and was modeled with a spinel structure in which all Mg^2+^ refined to the 16d site (Figure S6).

Building upon our discovery of the spinel Li_2_Mg_1/3_Zr_1/3_Cl_4_, we prepared the solid solution along the Li_2_MgCl_4_–Li_2_ZrCl_6_ series (Figure 4). This series can be represented by the chemical formula Li_2–*z*_Mg_1–3*z*/2_Zr_*z*_Cl_4_, with Li_2_MgCl_4_ (*z* = 0) and Li_2_ZrCl_6_ (*z* = 2/3) as the end members. Assuming that the excess charge of Zr^4+^ is compensated by Li^+^ vacancies, we hypothesized that this aliovalent substitution would improve ionic conductivity relative to the Li_2_MgCl_4_ end member^15^ and the Li_2_Mg_1/3_Zr_1/3_Cl_4_ spinel described above.

**Figure 3:**
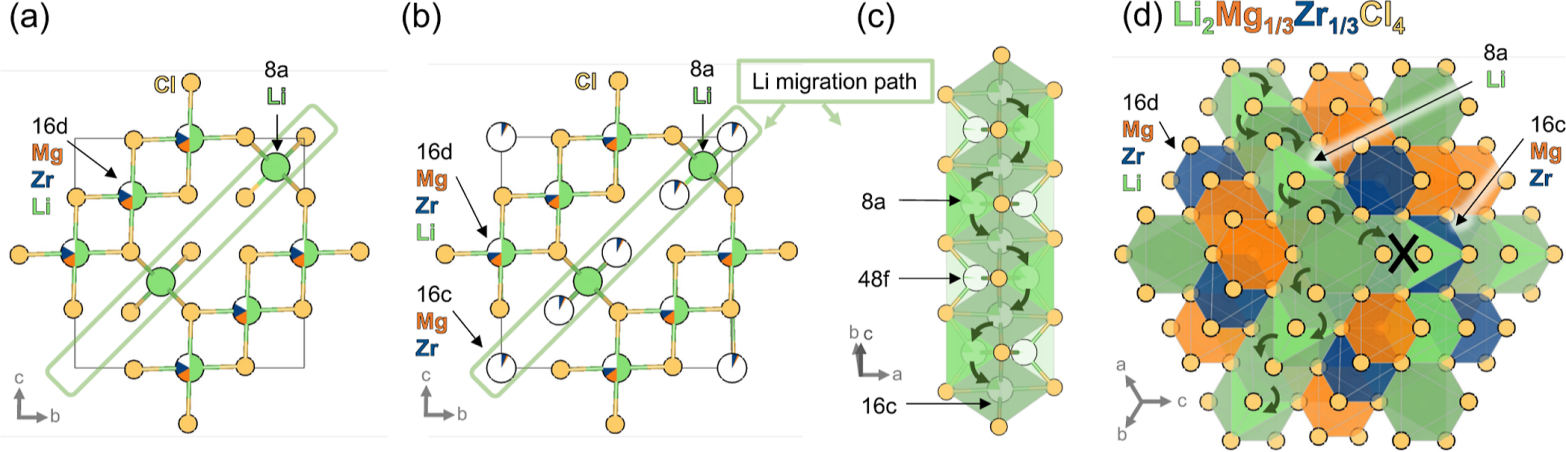
(a) Initial structural model of spinel Li_2_Mg_1/3_Zr_1/3_Cl_4_ showing Mg and Zr disorder on the 16d site compared with (b) the final structural model with Mg^2+^ and Zr^4+^ disorder on both the 16d and 16c sites (extracted from Rietveld fitting, Figure 1). (c) The Li-migration relies on hopping between tetrahedral 8a and 16c sites (with 48f as another possible site along this pathway). (d) A polyhedral representation of the Li_2_Mg_1/3_Zr_1/3_Cl_4_ structure shows how Mg^2+^/Zr^4+^ disorder on the 16c site may block some of the Li^+^ migration channels.

**Figure 4:**
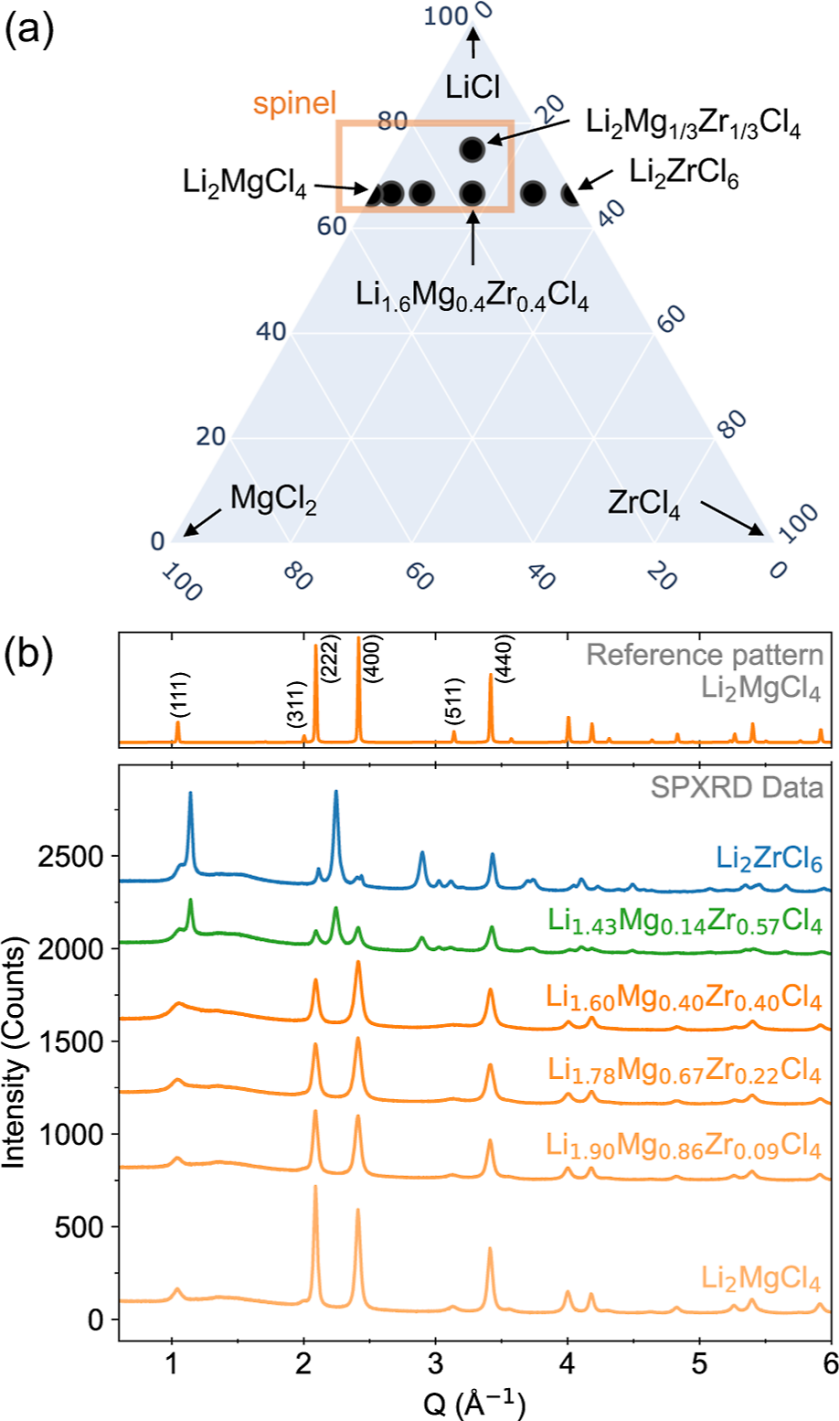
(a) Ternary phase diagram for the synthesized phases in the LiCl–MgCl_2_–ZrCl_4_ system. (b) SPXRD data for the series of materials synthesized along the Li_2_MgCl_4_–Li_2_ZrCl_6_ series, along with the simulated reference pattern for the spinel structure (Li_2_MgCl_4_).

SPXRD confirms that the spinel structure forms across the Li_2–*z*_Mg_1–3*z*/2_Zr_*z*_Cl_4_ series up to a maximum Zr content of Li_1.6_Mg_0.4_Zr_0.4_Cl_4_ (*z* = 0.4). Peak intensities of the spinel phase decrease with increasing Zr content, suggesting a decrease in crystallinity of the spinel phase (Figure 4b). Also, the relative intensities of peaks vary systematically, suggesting Zr-incorporation within the spinel structure. For example, the spinel (222) reflection decreases in intensity relative to the (400) reflection with increasing *z*. However, Mg^2+^ and Zr^4+^ are identical in size (0.72 Å radii),^20^ so the lattice parameter changes little across the series (Figure S8). At more Zr-rich compositions (*z* ≥ 0.57), a secondary phase forms that matches the Li_2_ZrCl_6_ pattern that has been previously reported.^21,22^ This phase has been described as isostructural with the Li_3_YCl_6_ structure (*P*3̅*m*1).^21,22^ LeBail refinements were effective at fitting the SPXRD data (Figures S7 and S8), but our attempts at Rietveld refinements on the Li_2_ZrCl_6_ phase were not satisfactory (Figure S9). Further discussion of this Li_2_ZrCl_6_ structure can be found in the Supporting Information. These data show that the spinel structure can tolerate a wide compositional range in the Li–Mg–Zr–Cl phase space, although a two-phase region is present for Zr-rich compositions.

### Electrochemical Characterization

**Figure 5:**
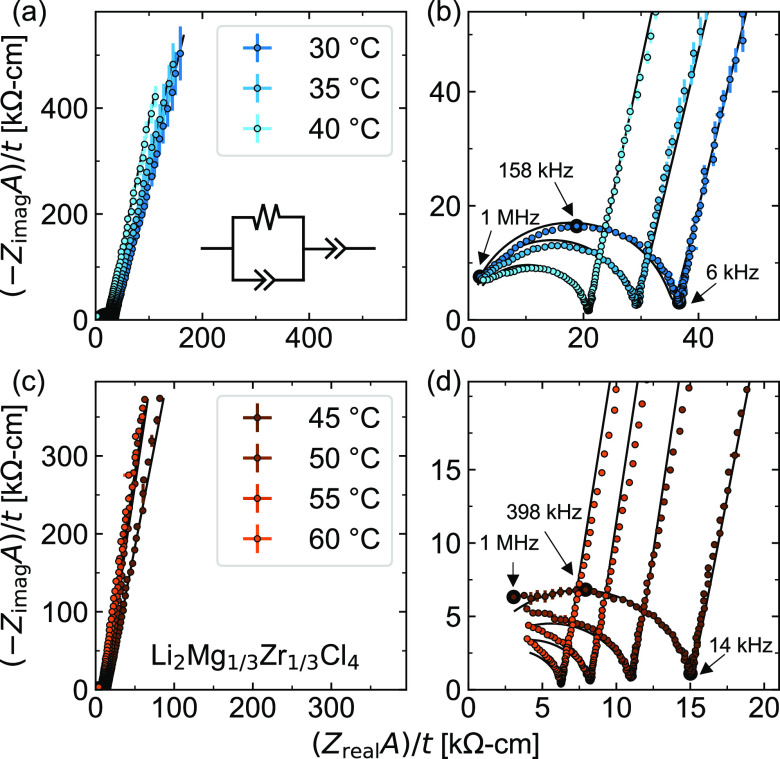
Nyquist plots as a function of temperature for the Li_2_Mg_1/3_Zr_1/3_Cl_4_ sample from 30 to 40 °C (a,b) and 45 to 60 °C (c,d). Points were averaged from three frequency sweeps, with error bars showing the standard deviations. Fits using the *R*_1_*Q*_1_ + *Q*_2_ circuit model (a, inset) are shown as black traces. Bode plots for the corresponding data are shown in Figure S12. Impedance was normalized by sample area and thickness. Select frequencies for the 30 and 45 °C measurements are marked with larger black circles.

EIS measurements were performed to assess the ionic conductivity of these new materials. We used carbon/electrolyte/carbon stacks to ensure consistent interfacial contact at low stack pressures (ca. 7 MPa, Figure S11).^23^ Nyquist plots of EIS data for Li_2_Mg_1/3_Zr_1/3_Cl_4_ show semicircles in the high-frequency region and near-linear tails in the low frequency region (Figure 5). We attribute this high-frequency semicircular feature to bulk ionic conductivity (σ_i_). However, the extracted capacitance values from this high-frequency feature (ca. 10^–10^ F) are higher than the value expected for pure bulk ionic conductivity (ca. 10^–12^ F).^24^ This may indicate that the high-frequency feature is a combination of bulk and grain boundary resistances, as previously observed in sulfide-based solid electrolytes.^25^ To avoid overfitting our data, we simply model this portion of the data with an *RQ* element, and use the corresponding resistance *R* to calculate σ_i_. The low-frequency tail is then modeled with an additional constant phase element (*Q*) for an overall *RQ* + *Q* equivalent circuit. For phases with σ_i_ > 0.1 mS/cm (e.g., Li_2_ZrCl_6_), this low-frequency tail exhibits curvature possibly indicative of charge transfer or nonblocking behavior of the carbon electrodes. For these phases, we use an *RQ* + *RQ* equivalent circuit. Additional fitting details are in the Supporting Information (Tables S3–S7 and Figures S13–S19).

**Figure 6:**
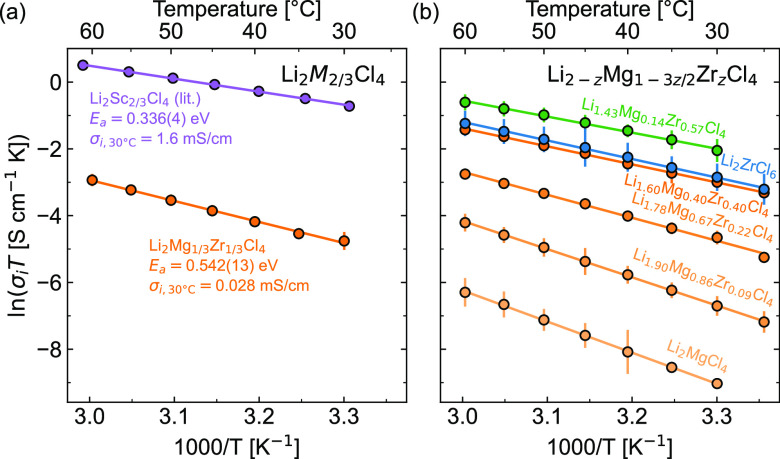
Arrhenius relationships between ionic conductivity (σ_i_) and temperature for (a) the Li_2_Mg_1/3_Zr_1/3_Cl_4_ spinel reported here compared with the Li_2_Sc_2/3_Cl_4_ spinel reported in literature by Zhou et al.^7^ and (b) the Li_2–*z*_Mg_1–3*z*/2_Zr_*z*_Cl_4_ series. Orange traces are for Mg-based spinel structures, while the green trace is the mixed spinel + Li_2_ZrCl_6_ material and the blue trace is Li_2_ZrCl_6_. The lines show the linear regression fits used to extract *E*_a_ values shown in Figure 7b.

Fits to the EIS spectra show that the Li_2_Mg_1/3_Zr_1/3_Cl_4_ spinel exhibits ionic conductivity of 0.028 mS/cm at 30 °C (Figure 6a). Therefore, Li_2_Mg_1/3_Zr_1/3_Cl_4_ has lower σ_i_ than the analogous spinel phases: Li_2_Sc_2/3_Cl_4_ (1.6 mS/cm at 30 °C)^7^ and Li_2_Sc_2/3–*x*_In_*x*_Cl_4_ solid solution (1.83 to 2.03 mS/cm at room temperature).^26^ Temperature-dependent EIS shows that Li_2_Mg_1/3_Zr_1/3_Cl_4_ exhibits an activation energy (*E*_a_) barrier of 0.542(13) eV (Figure 6). This barrier is higher than the barriers exhibited by Li_2_Sc_2/3_Cl_4_ and Li_2_Sc_2/3–*x*_In_*x*_Cl_4_ solid solution (ca. 0.330 eV).

The lower σ_i_ and higher *E*_a_ of Li_2_Mg_1/3_Zr_1/3_Cl_4_ compared to Sc-based spinel phases are likely related to structural differences. Li_2_Mg_1/3_Zr_1/3_Cl_4_ has a smaller lattice parameter than Li_2_Sc_2/3_Cl_4_ (Figure S8), and the narrower diffusion channels may raise *E*_a_ and decrease σ_i_.^27,28^ Alternatively, we propose that disorder in the Mg^2+^/Zr^4+^ cation sublattice may be detrimental to Li^+^ conduction pathways. As detailed in Figures 1 and 2, SPXRD suggests Mg and Zr in Li_2_Mg_1/3_Zr_1/3_Cl_4_ partially occupy the (normally vacant) 16c site, which may inhibit ionic conductivity by blocking migration pathways (Figure 3). Bond valence site energy (BVSE) calculations support this hypothesis (Figure S20). Neutron diffraction measurements of Li_2_Sc_2/3_Cl_4_ show that the Sc^3+^ partially occupies the 16d site, whereas the lithium ions are distributed across not only the 16d and 8a sites, but also the 16c and 48f sites.^7^ The high ionic conductivity of Li_2_Sc_2/3_Cl_4_ has been attributed to this additional Li^+^ site disorder,^7^ which may or may not be present in Li_2_Mg_1/3_Zr_1/3_Cl_4_ (neutron diffraction will be necessary to determine the Li distribution). Li_2_Sc_2/3_Cl_4_ may form in this way as a result of slow-cooling from the melt,^26^ which may allow the Sc^3+^ ions sufficient time and thermal energy to find their optimal site within the structure. Unfortunately, heat treatment of Li_2_Mg_1/3_Zr_1/3_Cl_4_ leads to phase separation (Figure S5), and we were not able to probe the impact of annealing on Mg^2+^ and Zr^4+^ cation (dis)ordering within the spinel structure. Lastly, if grain-boundary conductivity is substantially convoluted with bulk ionic conductivity for these Li–Mg–Zr–Cl spinels (as may be the case given the ∼10^–10^ F capacitance of the high frequency circuit element), then the true (but obscured) bulk ionic conductivity value may be more comparable with that of Li_2_Sc_2/3_Cl_4_.

**Figure 7:**
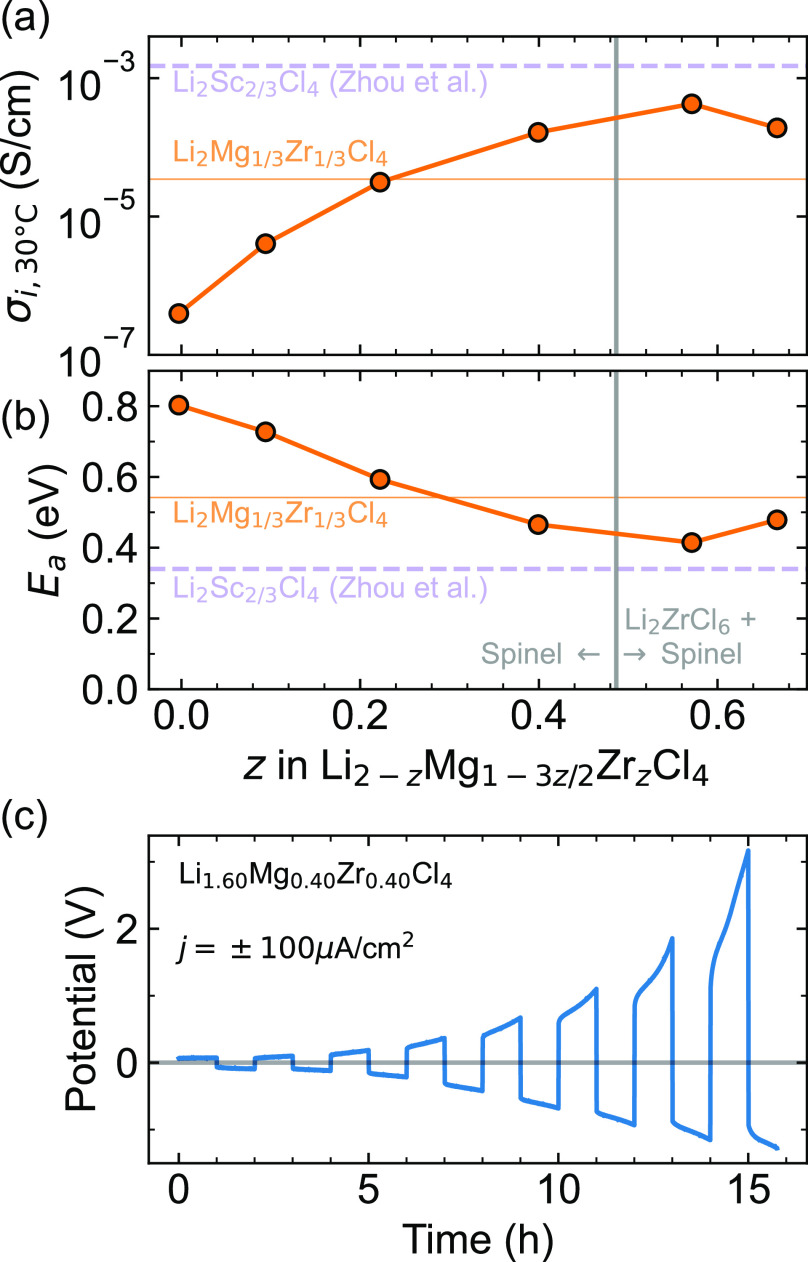
(a) Bulk ionic conductivity (σ_i_) values at 30 °C, extracted from EIS measurements on Li_2–*z*_Mg_1–3*z*/2_Zr_*z*_Cl_4_. (b) Activation energy (*E*_a_) for ion hopping extracted from temperature-dependent EIS (Figure 6). Statistical error bars are within the size of the markers. For comparison, values for Li_2_Mg_1/3_Zr_1/3_Cl_4_ from this work and Li_2_Sc_2/3_Cl_4_ from literature^7^ are included as horizontal lines. (c) Chronopotentiometry conducted on a Li/Li_1.60_Mg_0.4_Zr_0.4_Cl_4_/Li symmetric cell with an applied current density of *j* = ±100 μA cm^–2^ shows rapid degradation of the electrolyte.

Aliovalent substitution of Mg^2+^ by Zr^4+^ across the Li_2–*z*_Mg_1–3*z*/2_Zr_*z*_Cl_4_ series boosts ionic conductivity by 3 orders of magnitude for the spinel structure (Figure 7). EIS measurements show that Li_2_MgCl_4_ exhibits ionic conductivity near σ_i,30 °C_ = 4.0 × 10^–4^ mS/cm, but increasing Zr-substitution into Li_2–*z*_Mg_1–3*z*/2_Zr_*z*_Cl_4_ increases the ionic conductivity up to 0.23 mS/cm for the spinel structure (at *z* = 0.4). These values are similar to those recently reported for Li_1.67_Cr_0.33_Zr_0.33_Cl_4_ in the spinel structure (0.313 mS/cm at 30 °C),^29^ but still lower than the Li_2_Sc_2/3_Cl_4_ that inspired this work (1.6 mS/cm at 30 °C).^7^ The maximum conductivity is observed in the two-phase region with 0.43 mS/cm at *z* = 0.57. These changes in conductivity are inversely correlated with changes in *E*_a_ (Figure 7b), with *E*_a_ generally decreasing as *z* increases. These trends are consistent with prior literature on aliovalent substitution.^3^ For example, Zr^4+^ substitution for trivalent M^3+^ cations has been used to boost ionic conductivity in numerous Li_3–*x*_M_1–*x*_Zr_*x*_Cl_6_ phases (M = Y, Sc, Er).^30−33^ Here, we show that this strategy also works for divalent M^2+^ metals. Notably, Li_2_ZrCl_6_ exhibits both a lower σ_i_ and higher *E*_a_ than the Li_1.42_Mg_0.14_Zr_0.57_Cl_4_ (*z* = 0.57) composite, which contains both the Li_2_ZrCl_6_ and the spinel structures. These findings suggest that the disorder created in this quaternary system may contribute to the enhanced ionic conductivity relative to the ternary end members (i.e., Li_2_MgCl_4_ and Li_2_ZrCl_6_). As the *RQ* feature of our EIS models may include both bulk and grain boundary conductivities, the heterogeneous interfaces between the spinel and Li_2_ZrCl_6_ phases may contribute to this final boost in ionic conductivity.

Through chronopotentiometry experiments, we further find that the Li–Mg–Zr–Cl spinel electrolytes exhibit limited stability with lithium metal anodes (Figure 7c). Chronopotentiometry conducted on a Li/Li_1.60_Mg_0.4_Zr_0.4_Cl_4_/Li symmetric cell shows a rapid increase in potential after less than 16 h of cycling at ±100 μA/cm^2^ current density. Most likely, the Li metal reduces the Zr^4+^ to Zr^3+^ (or Zr) to create a nonpassivating interface.^3,34^ This behavior is not surprising, as chloride-based electrolytes are known to be unstable against lithium metal.^3,4,7^ The cathodic and full-cell stability of this class of materials will be the subject of future work.

In sum, this work demonstrates that II–IV substitution for III metals may be a promising strategy for discovering new lithium-ion conductors. The successful synthesis of Li_2_Mg_1/3_Zr_1/3_Cl_4_ and Li_2–*z*_Mg_1–3*z*/2_Zr_*z*_Cl_4_ in the spinel structure show the wide compositional tolerance of this phase. Therefore, further substitution may be possible, such as with other cheap and abundant metals like Zn^2+^, Ca^2+^, or Ti^4+^.^11^ How these substitutions would affect the (spinel) structure and properties remain open questions. Answering these questions could help unlock cost-effective solid electrolytes for safe and energy-dense all solid-state lithium-ion batteries.

## Conclusions

We report a new family of earth-abundant metal chloride spinel solid-state electrolytes. The inverse spinel Li_2_Mg_1/3_Zr_1/3_Cl_4_ and several members of the aliovalent substitution series Li_2–*z*_Mg_1–3*z*/2_Zr_*z*_Cl_4_ were prepared by high energy ball-milling. Although Li_2_Mg_1/3_Zr_1/3_Cl_4_ adopts an analogous structure to the previously reported Li_2_Sc_2/3_Cl_4_ superionic conductor, it exhibits lower ionic conductivity (ca. 10^–2^ mS/cm) than the Sc analog (>1 mS/cm), possibly owing to detrimental Mg and Zr cation disorder that may block Li^+^ migration pathways. Aliovalent substitution across the Li_2–*z*_Mg_1–3*z*/2_Zr_*z*_Cl_4_ series shows increased ionic conductivity of >0.1 mS/cm for *z* ≥ 0.4, suggesting that further optimization could realize superionic conductivity in this Li–Mg–Zr–Cl phase space. Most significantly, this work demonstrates a promising strategy for designing solid electrolytes comprised of inexpensive and earth-abundant chemistries: replace expensive trivalent metals (e.g., Sc^3+^, Y^3+^, In^3+^) with a mix of divalent and tetravalent metals (e.g., Mg^2+^, Ca^2+^, or Zn^2+^ with Ti^4+^ or Zr^4+^). This study clearly demonstrates that halide-based solid electrolytes can tolerate a wide range of cationic charges within the same crystal structure, and that these materials have immense potential for tuning composition to optimize for low cost and high performance.

## Experimental Section

### Synthesis

As the precursors and products are moisture sensitive, all materials were prepared in an argon glovebox and protected from oxygen and moisture during characterization. Phases in the LiCl–MgCl_2_–ZrCl_4_ system were synthesized by ball-milling in a Fritsch Pulverisette 7 Premium using 45 mL zirconia jars with 5 mm diameter zirconia milling balls (80 g total mass of milling balls). LiCl (Sigma-Aldrich, 99.9%, anhydrous), MgCl_2_ (Sigma-Aldrich, 99.99%, AnhydroBeads), and ZrCl_4_ (Thermo Fisher Scientific, 98%, anhydrous, contains 1–2% HfCl_4_) precursors were loaded into the jars following the stoichiometric ratios detailed in the text (ca. 5 g total mass). Samples were milled for 50 cycles of 10 min at 500 rpm followed by a 2 min rest (10 h total milling time). Annealing experiments were conducted by first pelletizing samples in a 6 mm diameter steel die using a hydraulic press (ca. 1 MPa uniaxial pressure) and then flame sealing the pellets in quartz ampules under vacuum (<30 mTorr) without air exposure.

### Structural Characterization

Laboratory powder X-ray diffraction (PXRD) was collected using a Rigaku Ultima IV diffractometer with a Cu Kα source. Samples were prepared for measurement on a zero-background silicon wafer and protected from atmosphere using polyimide tape. Samples were prepared for SPXRD by loading powders into quartz capillaries (0.3 mm OD, 0.29 mm ID) and flame sealing under vacuum. SPXRD measurements were collected at beamline 2–1 of the Stanford Synchrotron Radiation Lightsource (SSRL) at SLAC National Laboratory via the mail-in program (λ = 0.729487 Å).^35^ LeBail refinements were performed with Topas v6. Rietveld refinements were performed with GSAS II.^36^

### Electrochemical Characterization

EIS measurements were conducted on pelletized samples contained within custom insulating polyethyl ether ketone (PEEK) cell bodies (0.5 in inner diameter). Approximately 100 to 200 mg of powder was pelletized between two steel rods for at least 3 min (340 MPa uniaxial pressure), such that the pellet was at the center of the PEEK cell body (pellet thicknesses ca. 1 mm, see Table S3). Pellet densities were estimated to be 60 to 70% of theoretical based on crystallographic densities. To maintain electrical contact at low-stack pressures, we employed carbon black contacts (Figure S11).^23^ After pressing the pellet, one rod was removed, carbon black was added to coat the exposed face of the pellet (ca. 8 mg; TimCal C65), and the rod was reinserted. The process was repeated on the other side of the cell to create a steel/carbon/electrolyte/carbon/steel stack, which was then pressed again at 340 MPa uniaxial pressure for at least 3 min. The cell was sealed with o-rings prior to removal from the glovebox for temperature-dependent EIS.

Temperature-dependent EIS experiments were conducted between 30 and 60 °C. The sample was held in a custom spring jig at 7 MPa uniaxial pressure as measured by an in-line load cell. The carbon contacts ensured negligible changes in EIS spectra as a function of pressure between 0 and 9 MPa. The samples were heated in an oven to 60 °C overnight (ca. 12 h) and measured at 5 °C increments on cooling. The system was allowed to stabilize for 1 h at each temperature, after which 3 EIS sweeps were collected (1 MHz to 1 Hz, then 1 Hz to 1 MHz, then 1 MHz to 1 Hz again; 25 points/decade). These three spectra were averaged for each temperature, and modeled using *RQ* + *Q* or *RQ* + *RQ* equivalent circuits using custom Python software. Error bars on the ionic conductivity values and Arrhenius relationships were propagated from estimated error in cell dimensions and from statistical uncertainties in the EIS fits. Data are displayed in the frequency range of 1 MHz to 10 Hz, as the data at <10 Hz exhibited large statistical error bars.

Chronopotentiometry experiments were conducted in Li/electrolyte/Li symmetric cells using Li_1.60_Mg_0.4_Zr_0.4_Cl_4_ as the solid electrolyte. A freestanding pellet of the chloride was first pressed within the PEEK cell body (0.54 mm thickness, 1.27 cm^2^ area) using a hydraulic press 340 MPa uniaxial pressure for 3 min. Punches of Li foil (40 μm, on Cu) were then pressed into each side of the pellet (ca. 70 MPa). Chronopotentiometry measurements were conducted in 8 cycles of 100 μA/cm^2^ applied current density for 1 h followed by −100 μA/cm^2^ for another hour.

## Supporting Information Available

Precursor costs for chloride-based solid electrolytes, additional diffraction details, additional EIS details, and BVSE calculations (PDF)
Structure of ball-milled Li_2_Mg_1/3_Zr_1/3_Cl_4_ (CIF)
Structure of ball-milled Li_2_MgCl_4_ (CIF)
Structure of ball-milled + heat-treated Li_2_MgCl_4_ (CIF)

## Supplementary Material

cm4c01160_si_001.pdf

cm4c01160_si_002.cif

cm4c01160_si_003.cif

cm4c01160_si_004.cif
