# Supplementary material for: Expanding the Phase Space for Halide-Based Solid Electrolytes: Li–Mg–Zr–Cl Spinels
Source: Chem Mater. 2024 Jul 26;36(15):7283–91. doi: 10.1021/acs.chemmater.4c01160 (PMC11325553; doi:10.1021/acs.chemmater.4c01160)
Supplement: Supplementary file 1 — cm4c01160_si_001.pdf [file cm4c01160_si_001.pdf]

Supporting Information for: Expanding the phase space for halide-based solid electrolytes: Li-Mg-Zr-Cl spinels

Christopher L. Rom,*,† Philip Yox,‡ Abby M. Cardoza,‡ Rebecca W. Smaha,† Maxwell Q. Phan,† Trevor R. Martin,† and Annalise E. Maughan*,†,‡

†*National Renewable Energy Laboratory, Golden, Colorado 80401, United States*

‡*Department of Chemistry, Colorado School of Mines, Golden, Colorado 80401, United States*

E-mail: christopher.rom@nrel.gov; amaughan@mines.edu

Contents

Precursor costs for chloride-based electrolytes **S1**

Additional diffraction details **S2**

Additional EIS details **S12**

BVSE calculations **S25**

Precursor costs for chloride-based electrolytes

The precursors for $Li_{2-z}Mg_{1-3z/2}Zr_zCl_4$ electrolytes are substantially cheaper than those for $Li_2Sc_{2/3}Cl_4$ and $Li_2In_{2/3}Cl_4$ (Figure S1, Table S1). These estimates were based on publicly available prices for research-scale quantities of high-purity precursors (Table S1). Cost reductions are primarily driven by the low cost of $MgCl_2$ relative to $ScCl_3$ and $InCl_3$. This analysis suggests that optimization of ionic conductivity in the LiCl-$MgCl_2$-$ZrCl_4$ phase space could lead to economically competitive solid electrolytes for all-solid-state lithium batteries.

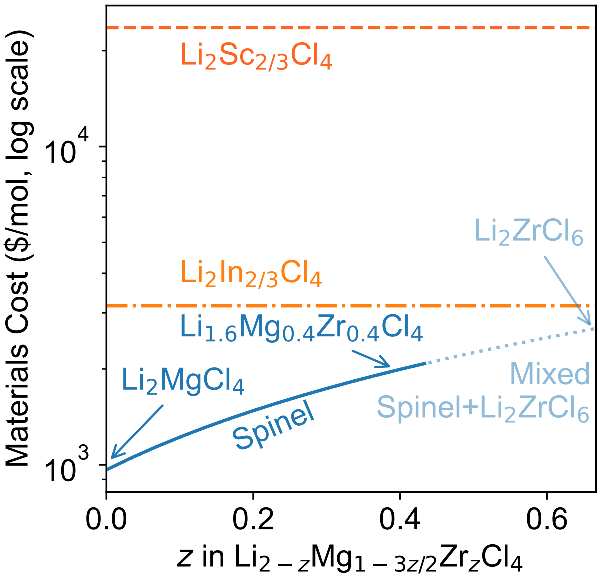

Figure S1: Cost estimates based on high purity, anhydrous precursors for $Li_{2-z}Mg_{1-3z/2}Zr_zCl_4$ electrolytes compared to analogous phases.

Table S1: Precursor costs from Sigma Aldrich, sourced on June 28, 2023 from https://www.sigmaaldrich.com/US/en. Precursor costs are based on the largest mass unit available for purchase at the specified purity. Anhydrous $InCl_3$ was not available at 99.99% purity, so the next closest option was used for these calculations.

**Precursor**	**Purity**	**$/gram**	**$/mol**	**Additional Details**
LiCl	99%	0.39	16.36	anhydrous, free-flowing, Redi-Dri™
$MgCl_2$	99.99%	9.78	931.16	AnhydroBeads™, 10 mesh
$ZrCl_4$	99.99%	17.12	3,989.64	anhydrous, powder
$ScCl_3$	99.99%	234.00	35,406.54	anhydrous, powder
$InCl_3$	99.999%	21.20	4,689.02	anhydrous, powder

Additional diffraction details

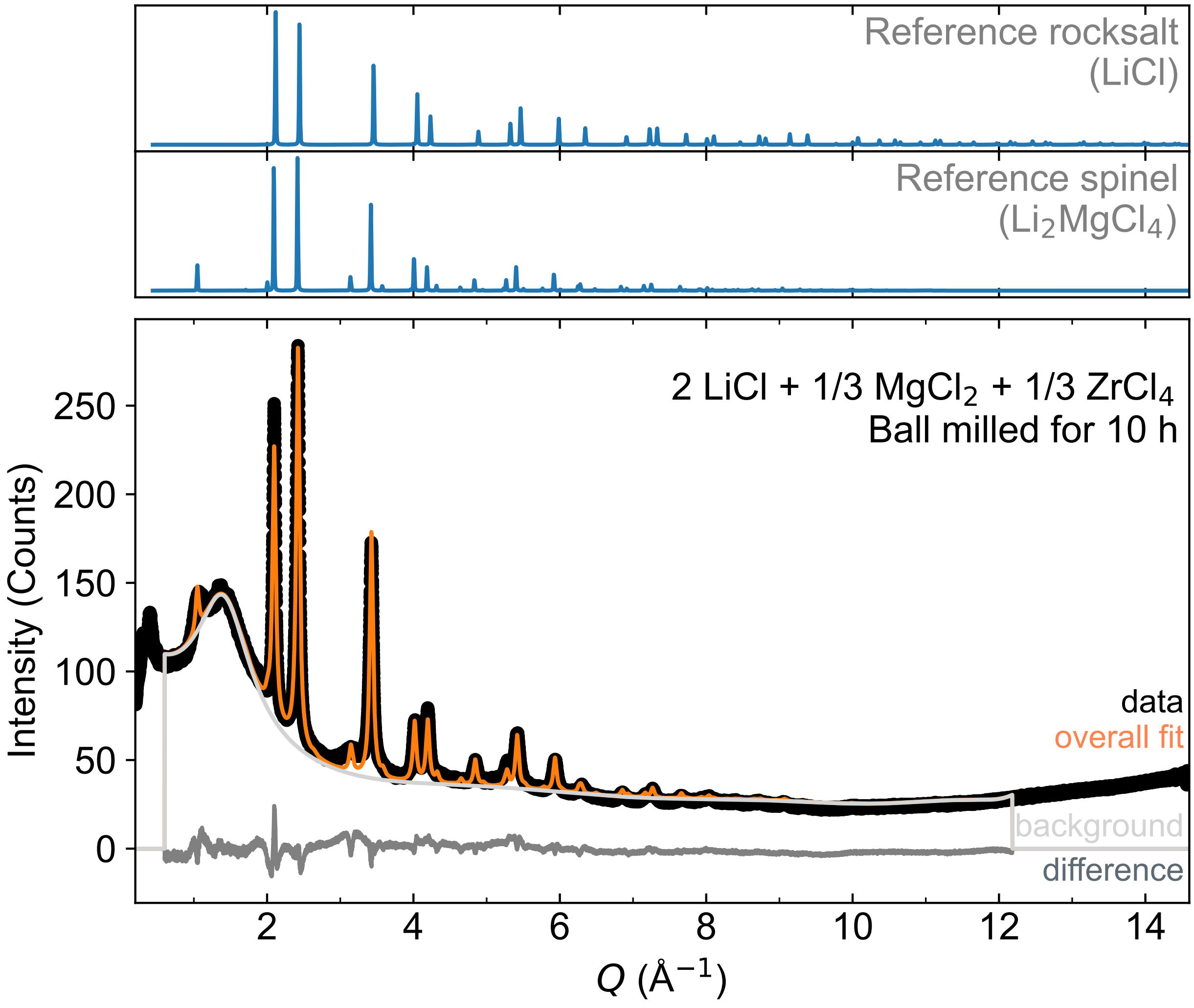

Figure S2: Diffraction pattern and Rietveld of the data shown in Figure 1b.

Figure S2 shows the full diffraction pattern of $Li_2Mg_{1/3}Zr_{1/3}Cl_4$ between $Q = 0.08$ Å^{-1} and $Q = 14.6$ Å^{-1}. The features below $Q < 1$ Å^{-1} were artifacts of the instrument or sample preparation (i.e., quartz capillaries nested in Kapton capillaries).

One alternative structure we considered in our Rietveld analysis of $Li_2Mg_{1/3}Zr_{1/3}Cl_4$ was the refinement of Mg^{2+} and Zr^{4+} exclusively on the 16*d* site but without the constraint that these two parameters be equivalent to one another. The results are shown in Figure S3 and Table S2. These results ($R_{wp} = 4.65\%$) are a slight improvement compared to the refinement in which Mg^{2+} and Zr^{4+} occupancies of the 16*d* site were constrained to one another ($R_{wp} = 5.10\%$, Figure 1a, Table 1). However, this refinement is still worse than the refinement in which both the 16*d* and 16*c* sites were considered ($R_{wp} = 3.89\%$, Figure 1b, Table 2).

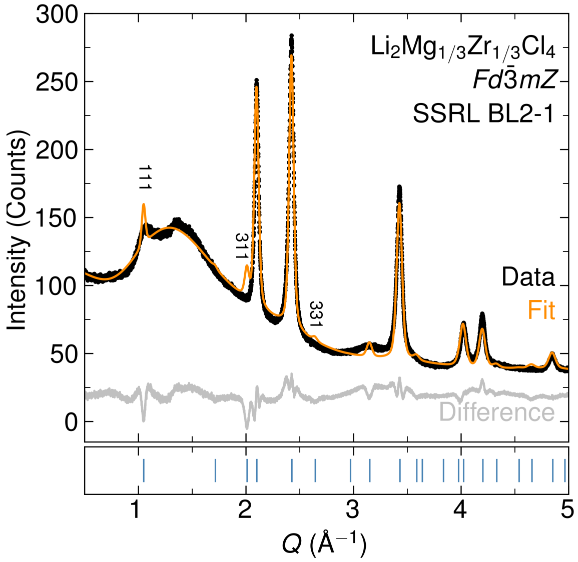

Figure S3: Diffraction pattern of $Li_2Mg_{1/3}Zr_{1/3}Cl_4$ with Rietveld refinement of Mg^{2+} and Zr^{4+} on the 16*d* site, unconstrained to one another.

Table S2: Results of the Rietveld refinement to SPXRD data of $Li_2Mg_{1/3}Zr_{1/3}Cl_4$ shown in Figure S3. Space group $Fd\bar{3}mZ$, $a = 10.3714(3)$ Å. $R_{wp} = 4.65\%$.

Site	Atom	x	y	z	occ.	$U_{11=22=33}$ (Å^2)	$U_{12=13=23}$ (Å^2)
8*a*	Li	0.125	0.125	0.125	1	0.04	–
16*d*	Li	0.5	0.5	0.5	0.5	0.04	–
16*d*	Mg	0.5	0.5	0.5	0.14(21)	0.0254(3)	0.024(7)
16*d*	Zr	0.5	0.5	0.5	0.15(6)	0.0254(3)	0.024(7)
32*e*	Cl	0.2520(1)	0.2520(1)	0.2520(1)	1	0.0245(3)	0.005(4)

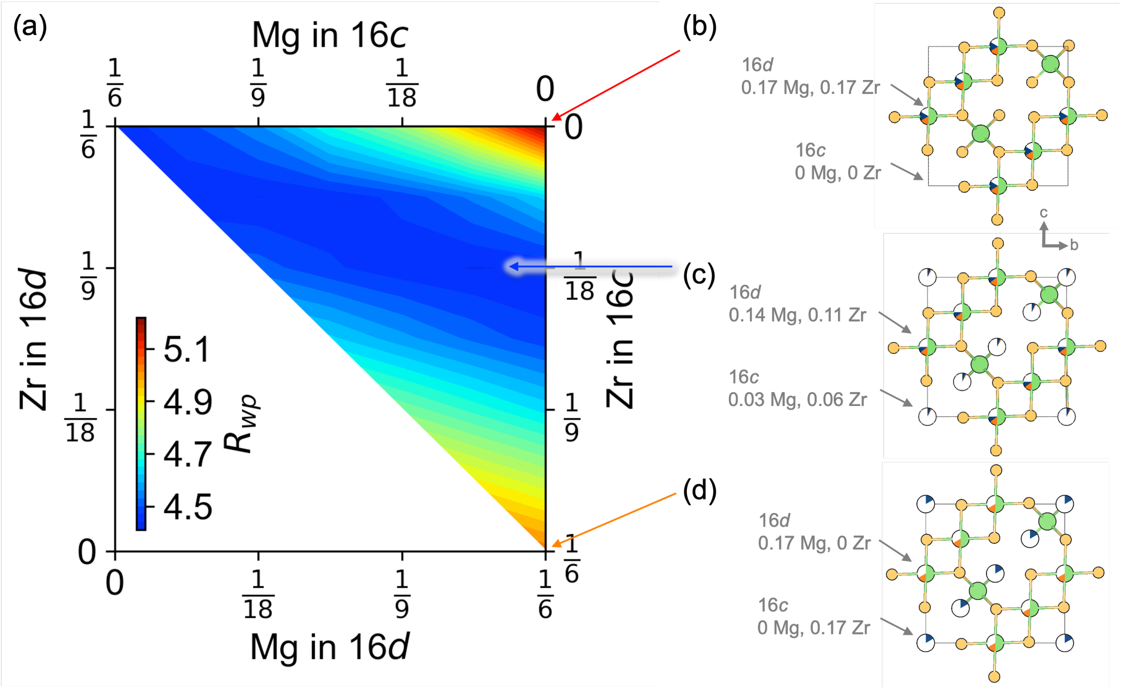

Figure S4: (a) Heatmap showing R_{wp} as a function Mg and Zr occupancy on the 16*d* and 16*c* sites, from parametric Rietveld refinements of SPXRD data from ball-milled $Li_2Mg_{1/3}Zr_{1/3}Cl_4$. Select structural visualizations of the $Li_2Mg_{1/3}Zr_{1/3}Cl_4$ spinel with (b) Mg and Zr exclusively occupying the 16*d* site, (c) Mg and Zr occupancy on both the 16*d* and 16*c* sites, and (d) Mg only on the 16*c* site and Zr only on the 16*d* site. Arrows indicate where these structures appear on the heat map.

To visualize the co-variance of the Mg and Zr occupancy in the SPXRD fits for ball-milled $Li_2Mg_{1/3}Zr_{1/3}Cl_4$, we conducted parametric Rietveld refinements (Figure S4). Site occupancies for the 16*d* and 16*c* sites were constrained such that total Mg and Zr occupancies were each 1/6 (i.e., 0.17) (the stoichiometry of $Li_2Mg_{1/3}Zr_{1/3}Cl_4$). Li occupancy was fixed at 0.5 for the 16*d* site and 1.0 for the 8*a* site. Other variables were allowed to freely refine (i.e., the lattice parameter *a*, size broadening, and atomic displacement parameters). The heatmap showing R_{wp} as a function of Mg and Zr occupancy on the 16*d* site (Figure S4a) indicates that a wide range of Mg and Zr occupancies provide similarly good fits ($R_{wp} < 4.5\%$). In contrast, the fit with Mg and Zr only on the 16*d* site (Figure S4b) is relatively poor ($R_{wp} > 5.1\%$). This analysis shows that the large uncertainty values for the Mg and Zr occupancies (Table 2) are a function of the co-variance between these two parameters. In other words, there is substantial uncertainty on the exact Mg and Zr content for the 16*c* site, but there is clearly a non-zero metal occupancy of this site.

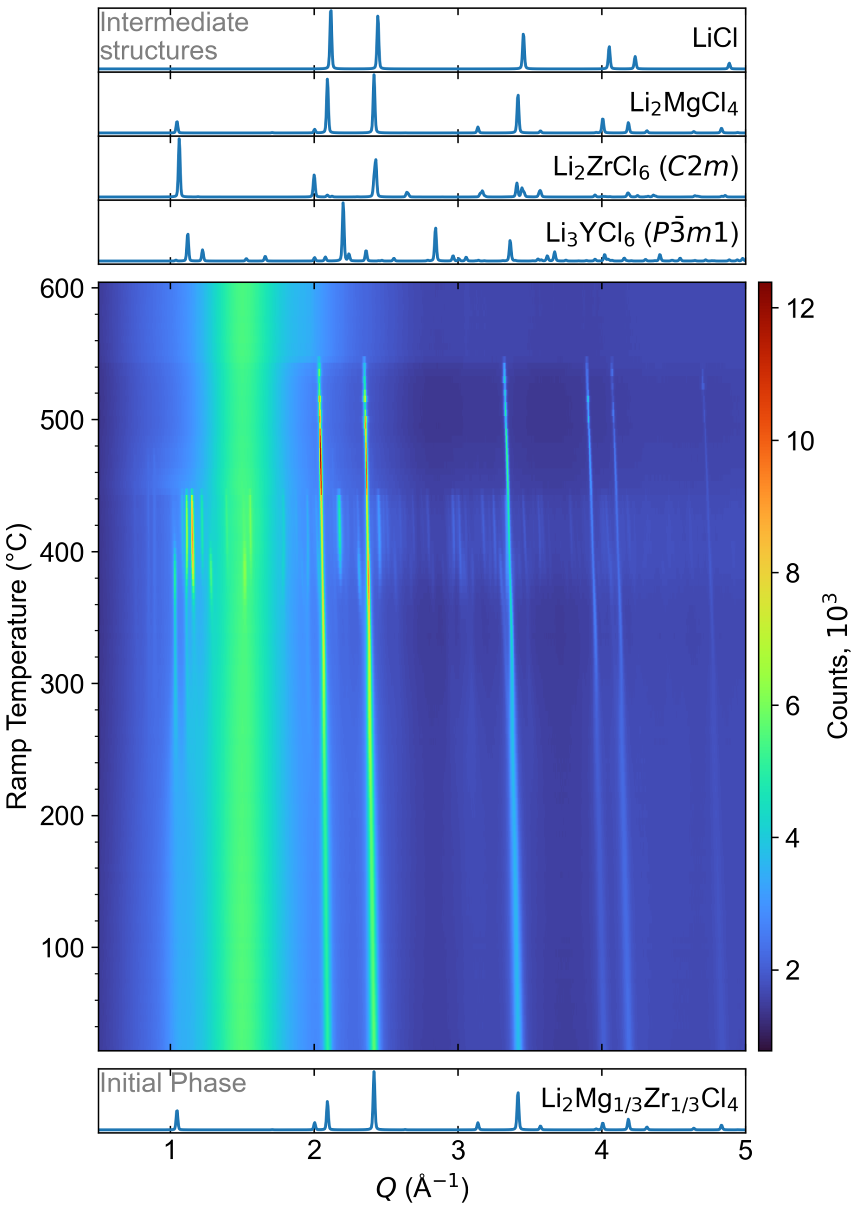

Figure S5: *In situ* SPXRD shows that the spinel structure of the ball-milled $Li_2Mg_{1/3}Zr_{1/3}Cl_4$ persists up to approximately 200 °C, above which the phase decomposes into Li_2MgCl_4, LiCl, and various polymorphs of Li_2ZrCl_6 (indexed to the Li_3YCl_6 ($P\bar{3}m1$) phase and the Li_2ZrCl_6 ($C2m$) phase).

To assess the crystallization pathway of ball-milled $Li_2Mg_{1/3}Zr_{1/3}Cl_4$ upon heat treatment, we conducted *in situ* SPXRD (Figure S5). *In situ* SPXRD measurements were conducted at beamline 17-BM-B of the Advanced Photon Source at Argonne National Laboratory ($\lambda = 0.24101$ Å). The ball-milled mixture of $Li_2Mg_{1/3}Zr_{1/3}Cl_4$ was loaded into a quartz capillary (0.7 mm OD, 0.69

mm ID), flame-sealed under vacuum, and placed in a flow-cell apparatus.[1] SPXRD frames were collected every 10 s followed by 20 s of deadtime as the sample was heated at rate of +10 °C/min up to 600 °C.

In situ SPXRD shows that $Li_2Mg_{1/3}Zr_{1/3}Cl_4$ phase separates at low temperatures (Figure S5). The initial spinel structure of the $Li_2Mg_{1/3}Zr_{1/3}Cl_4$ persists up to approximately 200 °C. Above 200 °C, peaks indexed to Li_2ZrCl_6 grow in. Between 380 °C and 450 °C, several phases are present: Li_2MgCl_4 and the Li_3YCl_6 polymorph of Li_2ZrCl_6. Between 450 °C and 550 °C, only a rocksalt structure is present, likely a cation disordered (Li,Mg,Zr)Cl rocksalt. However, this structure is likely Zr-poor, as *ex situ* reactions show that $ZrCl_4$ tends to volatize away above 450 °C. Above 550 °C, no crystalline peaks are observed, consistent with a molten phase. The spinel $Li_2Mg_{1/3}Zr_{1/3}Cl_4$ is not recovered on cooling; rather, LiCl and Li_2MgCl_4 are detected, consistent with $ZrCl_4$ volatization. We therefore conclude that $Li_2Mg_{1/3}Zr_{1/3}Cl_4$ has limited stability at elevated temperatures.

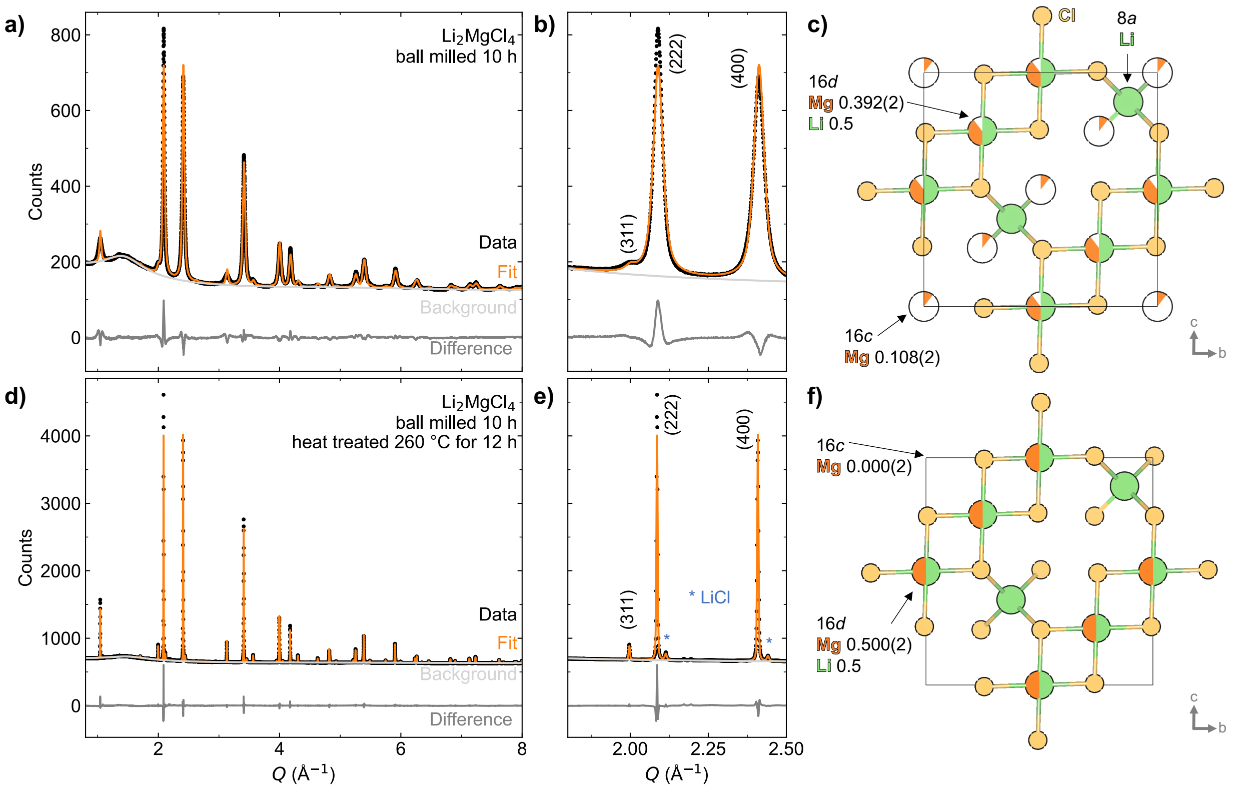

Figure S6: (a,b) Diffraction pattern and Rietveld analysis of a) ball-milled Li_2MgCl_4, with b) showing a focused region of the (311), (222), and (400) spinel reflections to highlight substantial peak broadening. c) The structure determined by Rietveld refinement with 0.108(2) Mg occupancy on the 16*c* site. Annealing the ball-milled Li_2MgCl_4 for 12 h at 260 °C leads to d,e) sharper peaks and f) all of the Mg refining to the 16*d* site.

The Li_2MgCl_4 does not decompose after heat treatment, but the Mg^{2+} cation disorder changes. Figure S6 shows SPXRD data and Rietveld analysis for ball-milled Li_2MgCl_4 (a-c) compared to the same sample after 12 h of heat treatment at 260 °C (d-f). With heat treatment, the Bragg peaks of the spinel phase sharpen, and a small LiCl secondary phase emerges (< 1 mol%). Rietveld refinements show that ball-milling induces some Mg^{2+} occupancy on the 16*c* site (ca. 20% of the Mg^{2+}, Figure S6c), but all of the Mg^{2+} refines to the 16*d* site after annealing (Figure S6f). Furthermore, the small uncertainty values for the Mg site occupancy (±0.002) demonstrate the distinct non-zero occupancy of the 16*c* site, and further supports the idea that the large uncertainty values in the occupancy (Table 2) stem from Mg/Zr co-variance. This analysis is consistent with prior work showing that ball-milling induces site disorder, whereas annealing increases ordering.[2]

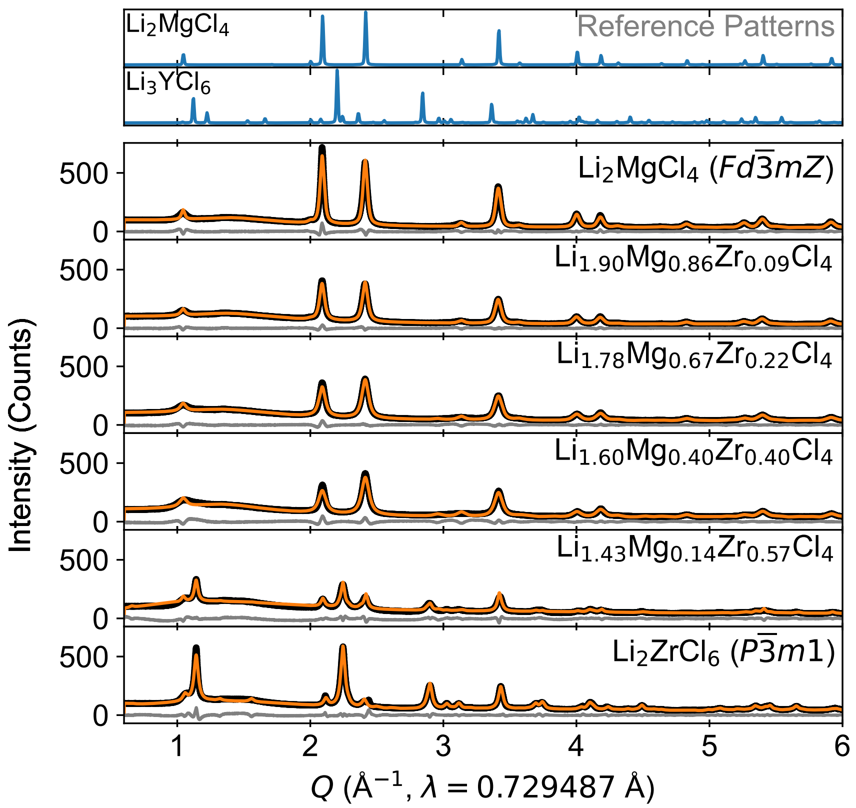

Figure S7: LeBail refinements on SPXRD data of the $Li_{2-z}Mg_{1-3z/2}Zr_zCl_4$ series.

LeBail refinements on the spinel phase show systematic trends in the lattice parameter, *a*, across the $Li_{2-z}Mg_{1-3z/2}Zr_zCl_4$ series (Figures S7 and S8). First, the ball-milled Li_2MgCl_4 has a larger *a* than the literature report of a single crystal prepared from a slow-cooled melt.[3] We hypothesize that this difference in lattice parameter may be related to disorder induced by ball-milling.[4] As Mg^{2+} is replaced with Zr^{4+} across the $Li_{2-z}Mg_{1-3z/2}Zr_zCl_4$ series, *a* initially expands, then gradually decreases with increasing Zr^{4+}-content. This decrease in *a* is consistent with decreasing Li^+ content with increasing *z*, as the Li-Cl bond length (2.57 Å)[5] is larger than the Mg-Cl and Zr-Cl bond lengths (both 2.49 Å),[6,7] based on the octahedral coordination in the binary chlorides.

The refined lattice parameters of the $Li_{2-z}Mg_{1-3z/2}Zr_zCl_4$ spinels are similar to those reported for Li_2MgCl_4 and $Li_2Sc_{2/3}Cl_4$ (Figure S8).[3,8] There is little variation in lattice parameter ($< 0.1\%$) for the $Li_{2-z}Mg_{1-3z/2}Zr_zCl_4$ compounds compared to the Li_2MgCl_4 reference compound.[3] The refined lattice parameter of $Li_2Mg_{1/3}Zr_{1/3}Cl_4$ is slightly smaller than the comparison structures (ca.

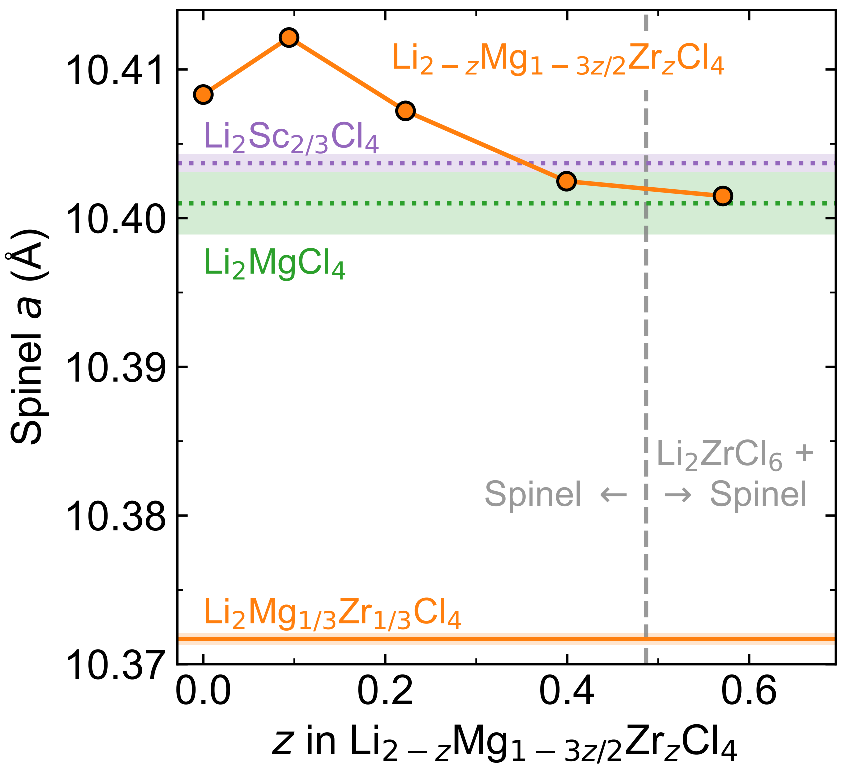

Figure S8: Lattice parameters from LeBail refinements for Li-Mg-Zr-Cl spinels (solid traces) compared to reference spinels from literature (dotted lines). Shaded regions around the horizontal lines represent statistical errors reported from the fits. Statistical error bars for the $Li_{2-z}Mg_{1-3z/2}Zr_zCl_4$ phases are within the size of the markers.

0.3 %). We take this as an indication that $Li_2Mg_{1/3}Zr_{1/3}Cl_4$ is more LiCl-like than the comparison structures (e.g., $Li_{1.6}Mg_{0.4}Zr_{0.4}Cl_4$). The equivalent spinel-like supercell for LiCl (rocksalt, $Fm\bar{3}m$, $a = 5.143$ Å)[5] would correspond to a lattice parameter of $a = 10.286$ Å. The differences in how Li-content affects the $Li_{2-z}Mg_{1-3z/2}Zr_zCl_4$ series and $Li_2Mg_{1/3}Zr_{1/3}Cl_4$ may be related to the differing Li:(Mg+Zr) ratios for these two sets of compounds (2:1 and 3:1, respectively). As unit cell volume and disorder are correlated with ionic conductivity,[9] these structural features have implications for that important property.

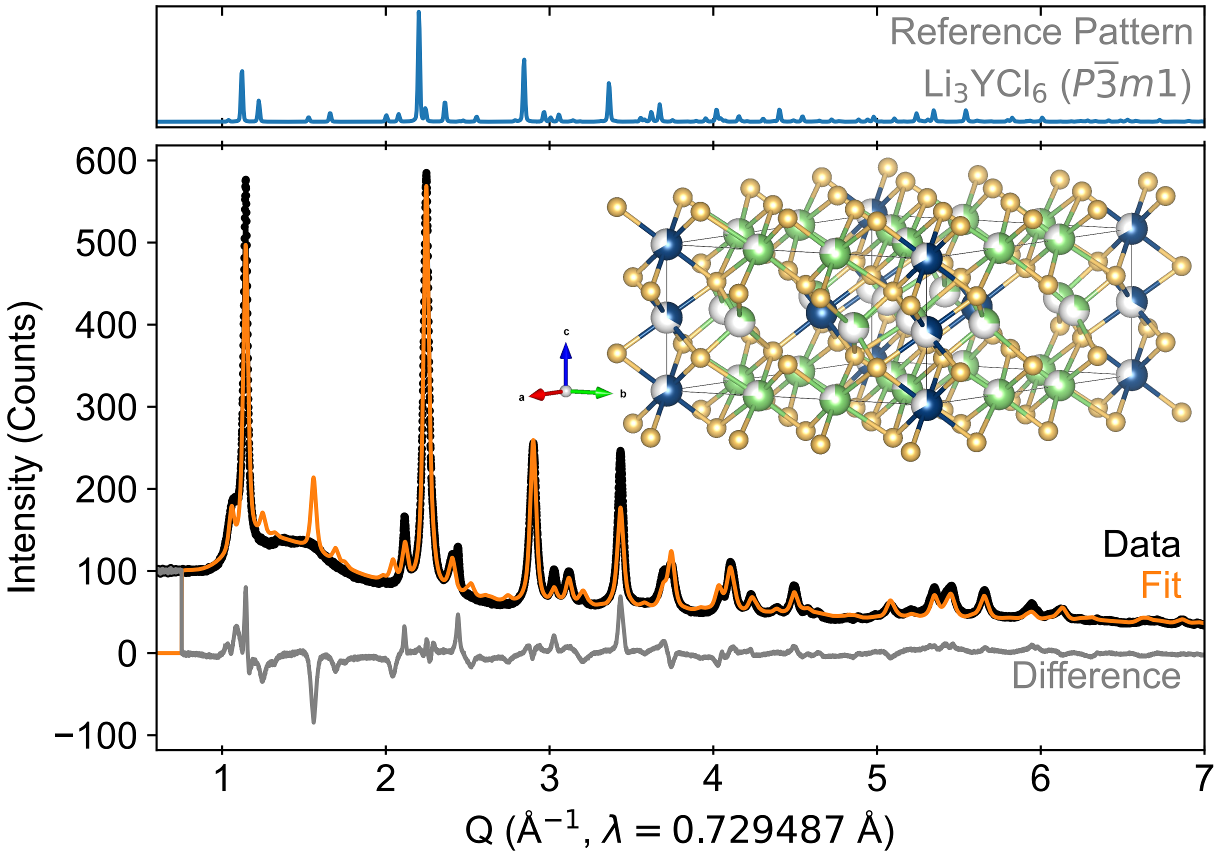

Figure S9: Rietveld refinement of the SPXRD data for the ball-milled Li_2ZrCl_6 sample. The fit (orange trace) deviates substantially from the data (black dots), suggesting that our model (inset) does not accurately describe the structure of the material.

We were not successfully able to model the structure of the Li_2ZrCl_6 from SPXRD data (Figure S9). Li_2ZrCl_6 has previously been prepared by ball-milling, and these prior reports describe this structure as isostructural with Li_3YCl_6 ($P\bar{3}m1$).[10,11] We were able to fit the SPXRD data for this phase using the LeBail method (Figure S7), which yielded lattice parameters of $a = 10.9835(2)$ Å and $c = 5.9295(2)$ Å. However, when we attempted to fit the data from our sample by the Rietveld method, our model produced peak intensities that do not match the data (Figure S9). This model was created by starting from the $P\bar{3}m1$ Na_2ZrCl_6 structure (ICSD #143651), and substituting Li^+ for the Na^+. Future work may require co-refinement of synchrotron PXRD and neutron diffraction to resolve this structure.

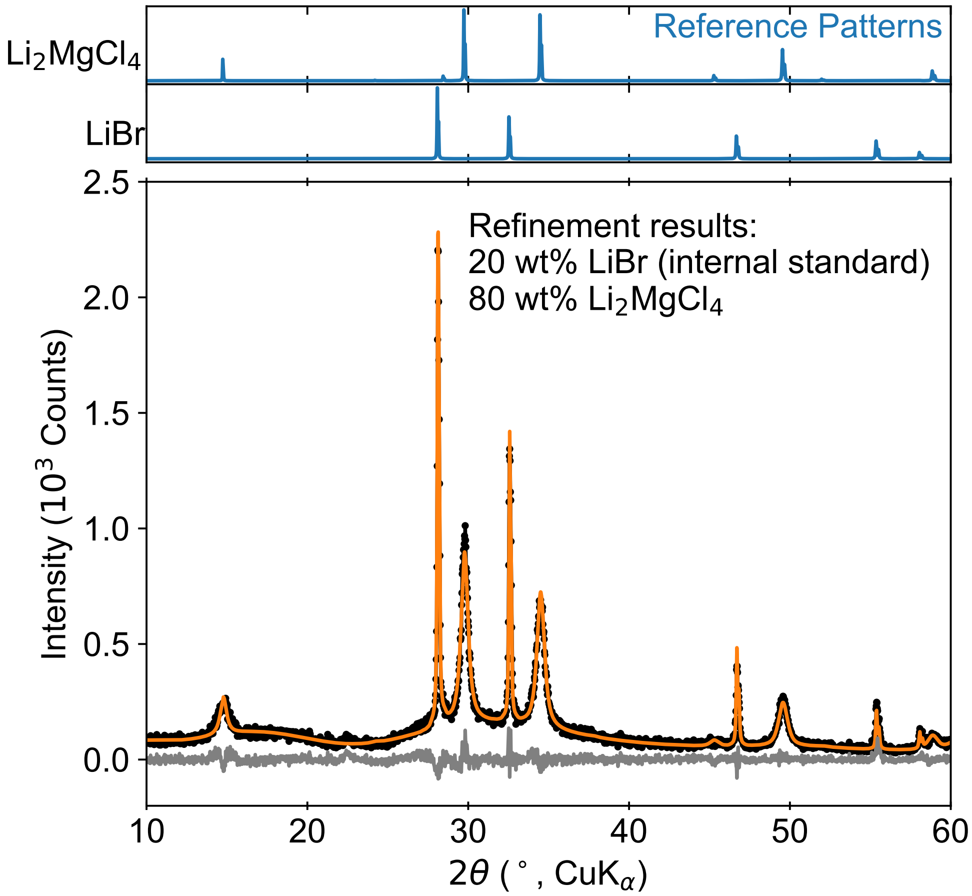

Figure S10: Quantitative phase analysis on ball-milled Li_2MgCl_4 with 20 wt% LiBr added as an internal standard.

To estimate the amount of amorphous material present in these samples, we performed quantitative phase analysis on the ball-milled Li_2MgCl_4 sample using LiBr as an internal standard (Figure S10). Data were collected using an air-free dome for a 20 minute collection time (longer collection times led to the appearance of hydrates in the pattern). Rietveld analysis with LiBr content fixed at the intentionally added value (20.0(4) wt%) reveals that Li_2MgCl_4 composes 80(1) wt% of this spiked sample, or 100(1) wt% of the original sample. This analysis implies that the amorphous content is below our detection limit. However, this analysis is limited by the short scan time and quantitative phase analysis is known to have a variation on the order of 3 to 4 wt%.[12]

Additional EIS details

Carbon contacts, as opposed to stainless steel (SS) contacts, were used for EIS measurements to ensure good electrical contact with the pellet under low stack pressures. When EIS measurements were conducted on SS/$Li_2Mg_{1/3}Zr_{1/3}Cl_4$/SS cell stacks, the high-frequency impedance increases dramatically when the pressure is decreased from 340 MPa to 1 MPa (Figure S11a and b, respectively). This increased impedance suggests decreased interfacial contact. In contrast, EIS measurements conducted after carbon black was applied to both sides of the pellet to form a SS/C/$Li_2Mg_{1/3}Zr_{1/3}Cl_4$/C/SS stack show similar high-frequency impedance at 340 MPa and 1 MPa stack pressure (Figure S11c and d, respectively). This consistency indicates that electrical contact is not substantially affected by stack pressure when carbon contacts are used.

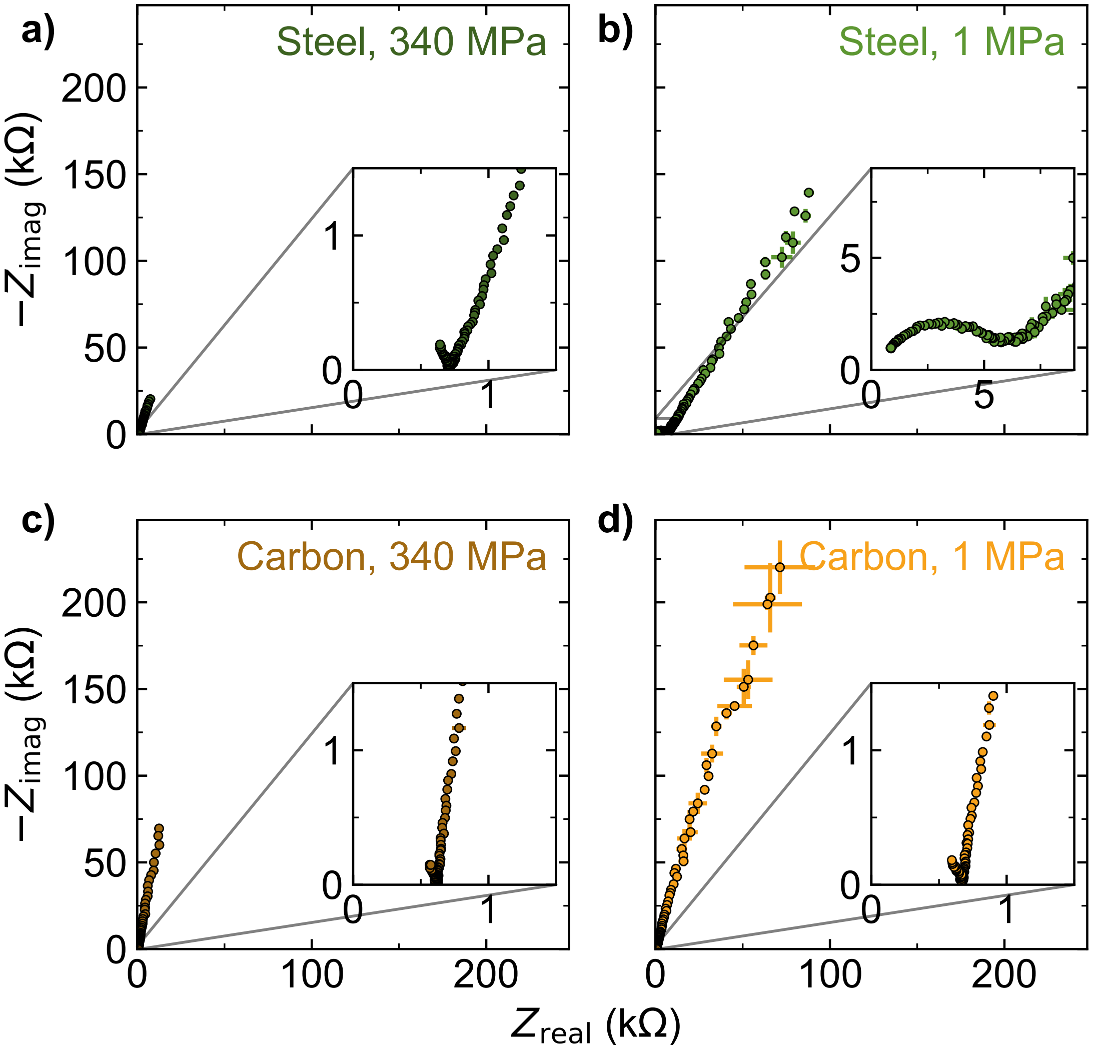

Figure S11: Nyquist plots for $Li_2Mg_{1/3}Zr_{1/3}Cl_4$ at room temperature as a function electrical contact and stack pressure: a) steel/$Li_2Mg_{1/3}Zr_{1/3}Cl_4$/steel contacts at 340 MPa and b) 1 MPa compared with the same pellet with carbon black contacts at c) 340 MPa and d) 1 MPa. The insets show the high-frequency region indicative of bulk ionic conductivity. The scales for insets in a, c, and d are identical.

Table S3 shows the dimensions of the pellets used for EIS measurements. Tables S4 and S5 show the fit values for EIS measurements at 30 °C as a function of composition. These R_1 resistance values and pellet dimensions (t = thickness, A = area) were used to calculate bulk ionic conductivity ($\sigma_i = \sigma_1 = \frac{t}{R_1 A}$). The constant phase element parameters (Q and n) and resistance of the corresponding RQ element were used to calculate capacitance (e.g., $C_1 = (Q_1 * R_1^{1-n_1})^{\frac{1}{n_1}}$).[13] These calculated values are shown in Tables S6 and S7.

Table S3: Cell dimensions used for EIS measurements of $Li_2Mg_{1/3}Zr_{1/3}Cl_4$ and $Li_{2-z}Mg_{1-3z/2}Zr_zCl_4$. The PEEK cell inner diameter was 1.270 ± 0.001 cm, leading to an uncertainty in the area of ± 0.0014 cm^2. Sample thicknesses were measured to an uncertainty of ± 0.001 cm.

**Stoichiometry**	z	**Thickness (cm)**	**Area (cm^2)**
$Li_2Mg_{1/3}Zr_{1/3}Cl_4$	-	0.059	1.267
Li_2MgCl_4	0	0.115	1.267
$Li_{1.90}Mg_{0.86}Zr_{0.09}Cl_4$	0.09	0.059	1.267
$Li_{1.78}Mg_{0.67}Zr_{0.22}Cl_4$	0.22	0.098	1.267
$Li_{1.60}Mg_{0.40}Zr_{0.40}Cl_4$	0.40	0.078	1.267
$Li_{1.42}Mg_{0.14}Zr_{0.57}Cl_4$	0.57	0.078	1.267
Li_2ZrCl_6	0.67	0.098	1.267

Table S4: Representative equivalent circuits parameters and error values from statistical uncertainty (shown for 30 °C) for the first circuit element (i.e., R_1Q_1) of the $R_1Q_1 + Q_2$ and $R_1Q_1 + R_2Q_2$ models used to fit EIS data from $Li_2Mg_{1/3}Zr_{1/3}Cl_4$ and $Li_{2-z}Mg_{1-3z/2}Zr_zCl_4$.

**Stoichiometry**	z	R_1 (Ω)	$R_{1,err}$ (Ω)	Q_1 (S/s^n)	$Q_{1,err}$ (S/s^n)	n_1
$Li_2Mg_{1/3}Zr_{1/3}Cl_4$	-	1.64E+03	4.76E+00	8.13E-10	1.19E-10	0.97
Li_2MgCl_4	0.00	2.29E+05	1.43E-08	4.35E-10	8.96E-11	0.95
$Li_{1.90}Mg_{0.86}Zr_{0.09}Cl_4$	0.09	1.15E+04	1.87E+01	5.24E-10	3.31E-11	0.99
$Li_{1.78}Mg_{0.67}Zr_{0.22}Cl_4$	0.22	2.47E+03	4.55E+00	9.71E-10	1.39E-11	0.91
$Li_{1.60}Mg_{0.40}Zr_{0.40}Cl_4$	0.40	3.74E+02	1.99E+00	7.35E-10	1.83E-10	0.97
$Li_{1.42}Mg_{0.14}Zr_{0.57}Cl_4$	0.57	1.75E+02	4.85E+00	2.75E-10	1.25E-09	1.00
Li_2ZrCl_6	0.67	4.12E+02	1.22E+00	7.41E-10	1.16E-10	0.97

Table S5: Representative equivalent circuits parameters and error values from statistical uncertainty (shown for 30 °C) for the second circuit element (i.e., Q_2 or R_2Q_2) of the $R_1Q_1+Q_2$ and $R_1Q_1+R_2Q_2$ models used to fit EIS data from $Li_2Mg_{1/3}Zr_{1/3}Cl_4$ and $Li_{2-z}Mg_{1-3z/2}Zr_zCl_4$.

**Stoichiometry**	z	R_2 (Ω)	$R_{2,err}$ (Ω)	Q_2 (S/s^n)	$Q_{2,err}$ (S/s^n)	n_2
$Li_2Mg_{1/3}Zr_{1/3}Cl_4$	-	-	-	1.16E-06	2.26E-08	0.85
Li_2MgCl_4	0.00	-	-	2.83E-07	6.27E-09	0.93
$Li_{1.90}Mg_{0.86}Zr_{0.09}Cl_4$	0.09	-	-	5.91E-07	2.53E-08	0.89
$Li_{1.78}Mg_{0.67}Zr_{0.22}Cl_4$	0.22	-	-	8.95E-07	1.65E-08	0.91
$Li_{1.60}Mg_{0.40}Zr_{0.40}Cl_4$	0.40	4.57E+04	3.29E+02	2.48E-06	1.00E-08	0.86
$Li_{1.42}Mg_{0.14}Zr_{0.57}Cl_4$	0.57	1.12E+05	4.43E+03	2.02E-06	2.11E-08	0.88
Li_2ZrCl_6	0.67	9.58E+04	3.62E-04	2.85E-06	7.71E-09	0.79

Table S6: Representative values for bulk ionic conductivity ($\sigma_1 = \sigma_i$) and high-frequency capacitance (C_1) calculated from the cell dimensions shown in Table S3 and EIS fits at 30 °C shown in Table S4. Uncertainty values (in parentheses) are propagated from statistical errors in the fit values and uncertainty in cell dimensions.

**Stoichiometry**	z	σ_1 (S/cm)	$\sigma_{1,err}$ (S/cm)	C_1 (F)	$C_{1,err}$ (F)
$Li_2Mg_{1/3}Zr_{1/3}Cl_4$	-	2.83E-05	1.60E-06	5.60E-10	8.20E-11
Li_2MgCl_4	0.00	3.96E-07	3.50E-09	2.76E-10	5.69E-11
$Li_{1.90}Mg_{0.86}Zr_{0.09}Cl_4$	0.09	4.05E-06	1.77E-07	4.81E-10	3.04E-11
$Li_{1.78}Mg_{0.67}Zr_{0.22}Cl_4$	0.22	3.14E-05	1.38E-06	2.59E-10	3.75E-12
$Li_{1.60}Mg_{0.40}Zr_{0.40}Cl_4$	0.40	1.64E-04	1.22E-05	4.38E-10	1.09E-10
$Li_{1.42}Mg_{0.14}Zr_{0.57}Cl_4$	0.57	4.25E-04	7.09E-05	2.75E-10	1.25E-09
Li_2ZrCl_6	0.67	1.88E-04	1.04E-05	4.38E-10	6.84E-11

Table S7: Representative values for grain boundary ionic conductivity (σ_2) and lower-frequency capacitance (C_2) calculated from the cell dimensions shown in Table S3 and EIS fits at 30 °C shown in Table S5. Uncertainty values (in parentheses) are propagated from statistical errors in the fit values and uncertainty in cell dimensions.

**Stoichiometry**	z	σ_2 (S/cm)	$\sigma_{2,err}$ (S/cm)	C_2 (F)	$C_{2,err}$ (F)
$Li_2Mg_{1/3}Zr_{1/3}Cl_4$	-	-	-	-	-
Li_2MgCl_4	0.00	-	-	-	-
$Li_{1.90}Mg_{0.86}Zr_{0.09}Cl_4$	0.09	-	-	-	-
$Li_{1.78}Mg_{0.67}Zr_{0.22}Cl_4$	0.22	-	-	-	-
$Li_{1.60}Mg_{0.40}Zr_{0.40}Cl_4$	0.40	1.35E-06	1.99E-08	1.73E-06	1.43E-08
$Li_{1.42}Mg_{0.14}Zr_{0.57}Cl_4$	0.57	5.52E-07	2.30E-08	1.64E-06	6.74E-08
Li_2ZrCl_6	0.67	8.08E-07	8.29E-09	2.00E-06	5.41E-09

Bode plots for EIS measurements of $Li_2Mg_{1/3}Zr_{1/3}Cl_4$ are shown in Figure S12. The minimum in the phase angle shifts to higher frequency with increasing temperature, consistent with faster ion hoping. Nyquist plots and corresponding fits for the $Li_{2-z}Mg_{1-3z/2}Zr_zCl_4$ series are shown in Figures S13-S18.

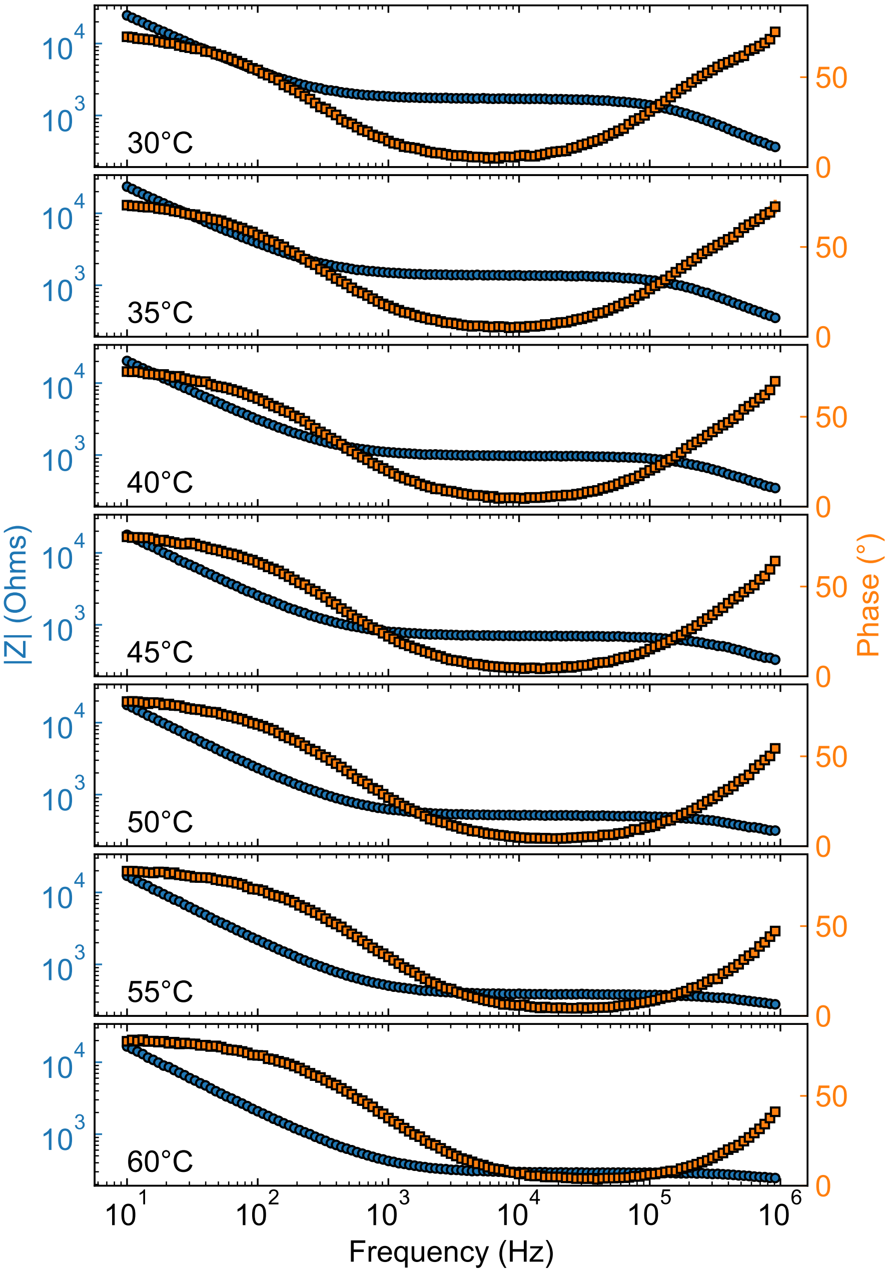

Figure S12: Bode plots as a function of temperature for the $Li_2Mg_{1/3}Zr_{1/3}Cl_4$ sample. Points were averaged from three frequency sweeps. Error bars of standard deviations are within the size of the markers. Nyquist plots for the corresponding data are shown in Figure 5.

Nyquist plots were fit using either $R_1Q_1 + Q_2$ or $R_1Q_1 + R_2Q_2$ equivalent circuit models. Phases with low ionic conductivity ($\sigma_i < 0.1$ mS/cm) were fit with the $R_1Q_1 + Q_2$ model (Figures 5 and S13-S15). For phases with high bulk ionic conductivity ($\sigma_i > 0.1$ mS/cm), curvature in the low-frequency region of the Nyquist plots were noticeable (Figures S16-S18). Therefore, we replaced the Q_2 element with a second R_2Q_2 element to fit this curvature, using an overall $R_1Q_1 + R_2Q_2$ equivalent circuit. This feature may indicate charge transfer resistance, as the carbon electrodes may exhibit non-blocking behavior.

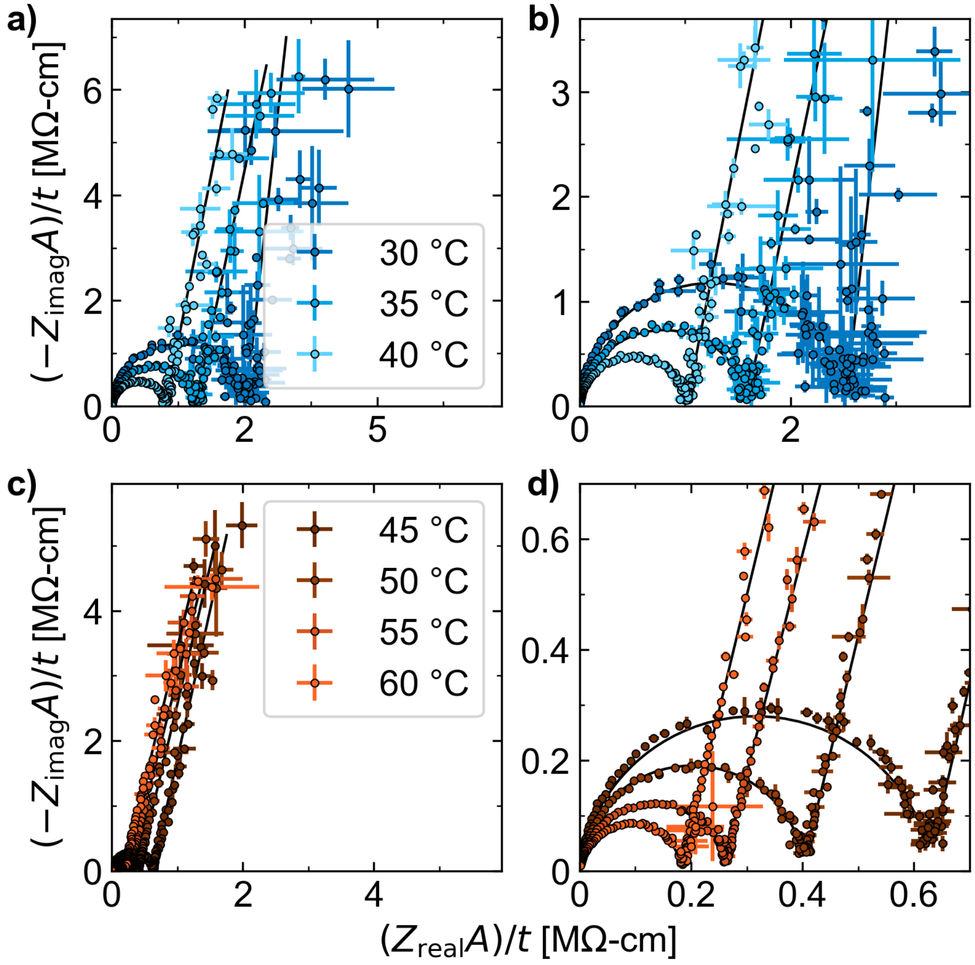

Figure S13: Nyquist plots for Li_2MgCl_4 as a function of temperature, fit with an $R_1Q_1 + Q_2$ equivalent circuit and normalized by sample area and thickness.

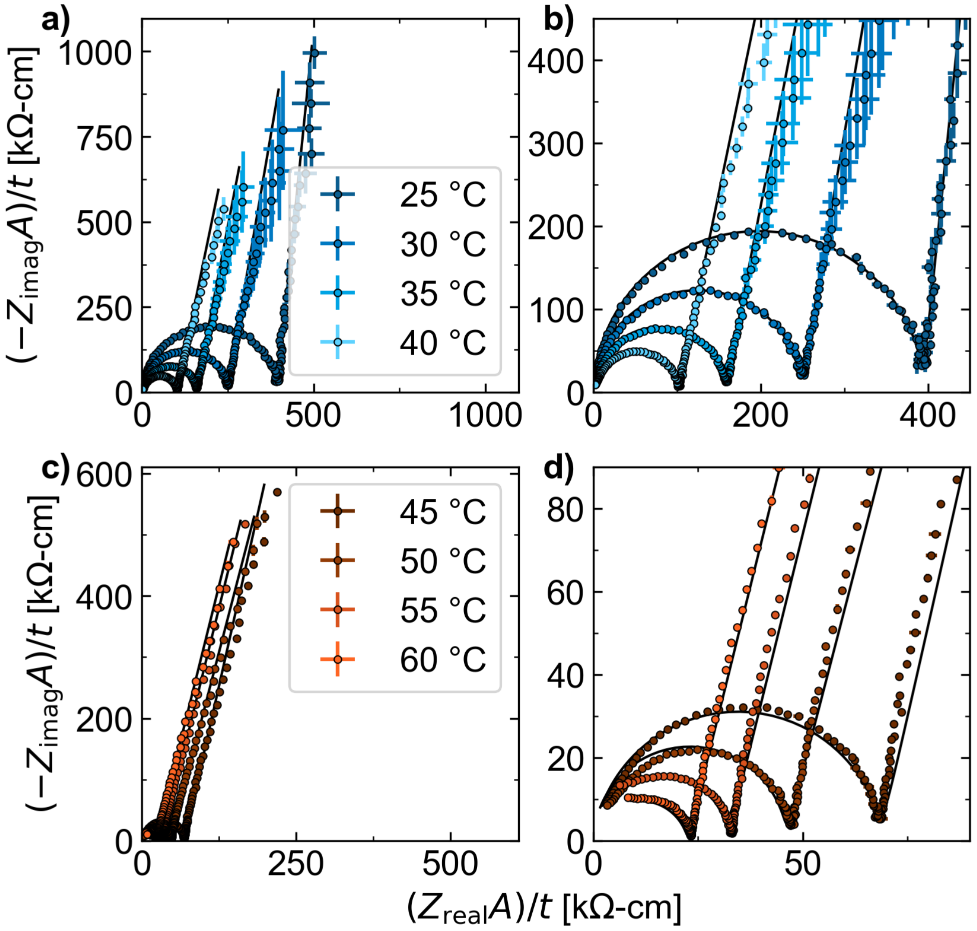

Figure S14: Nyquist plots for $Li_{1.90}Mg_{0.86}Zr_{0.09}Cl_4$ as a function of temperature, fit with an $R_1Q_1 + Q_2$ equivalent circuit and normalized by sample area and thickness.

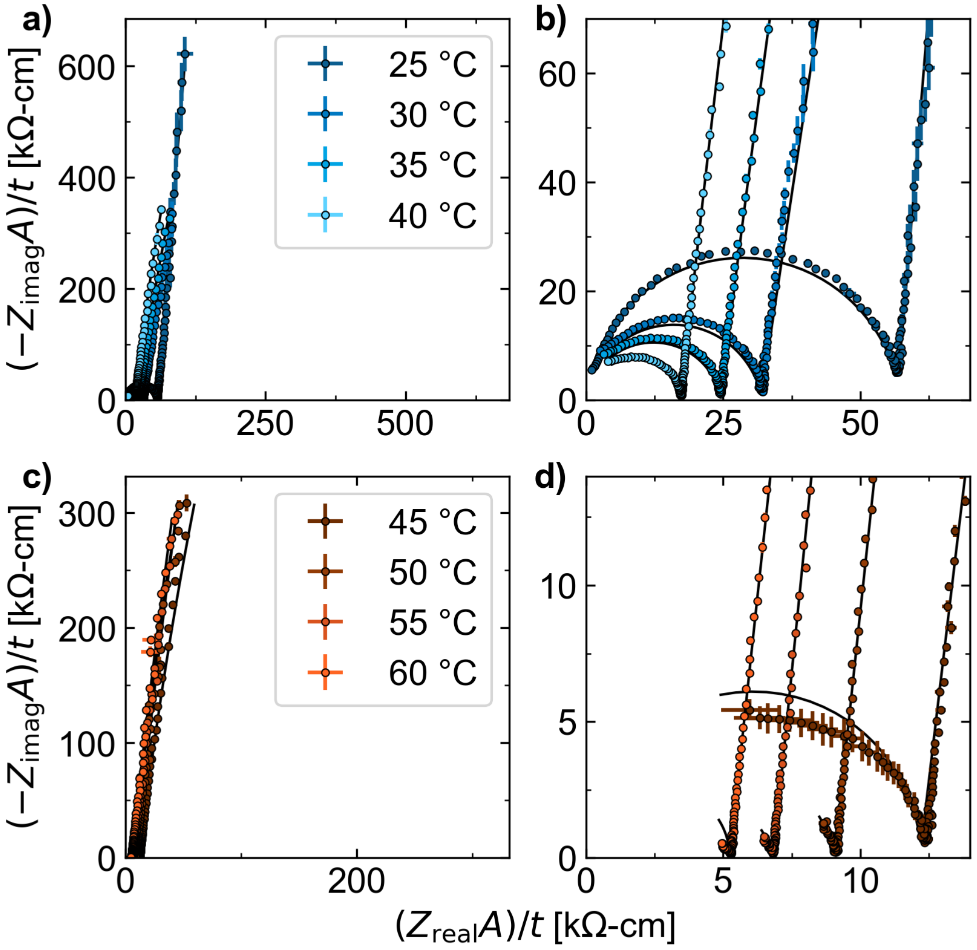

Figure S15: Nyquist plots for $Li_{1.78}Mg_{0.67}Zr_{0.22}Cl_4$ as a function of temperature, fit with an $R_1Q_1 + Q_2$ equivalent circuit and normalized by sample area and thickness.

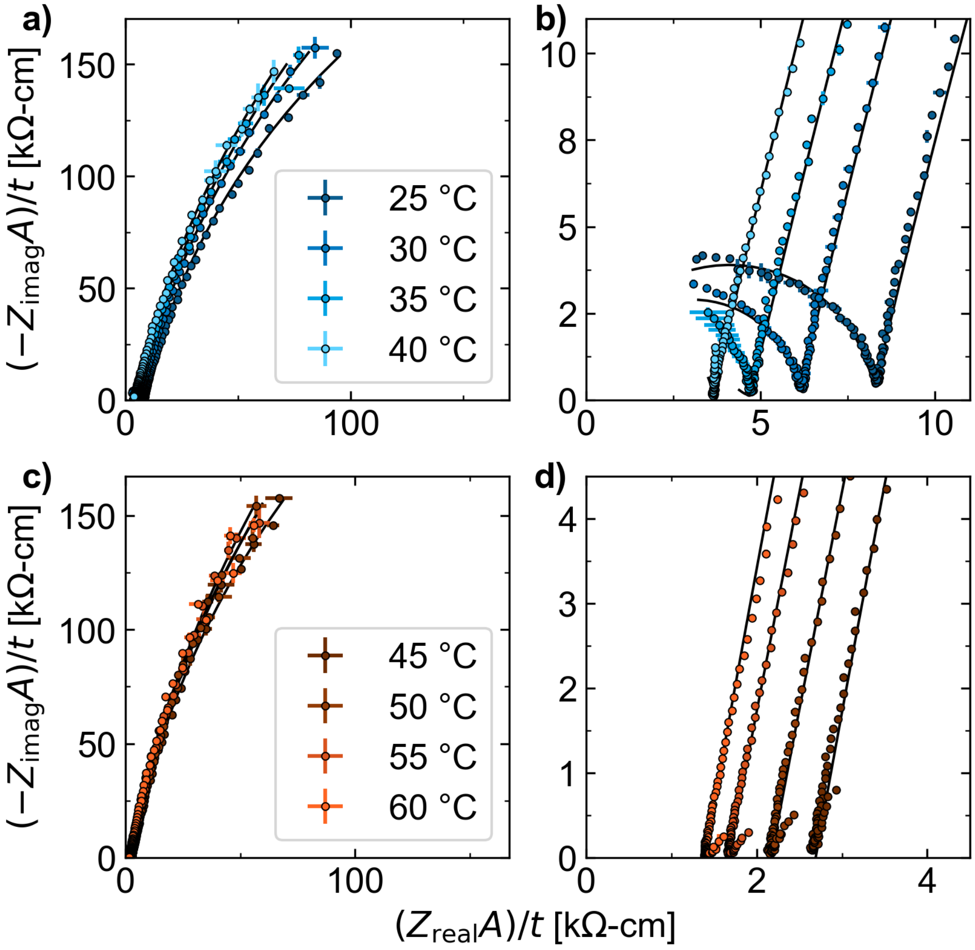

Figure S16: Nyquist plots for $Li_{1.60}Mg_{0.40}Zr_{0.40}Cl_4$ as a function of temperature, fit with an $R_1Q_1 + R_2Q_2$ equivalent circuit and normalized by sample area and thickness.

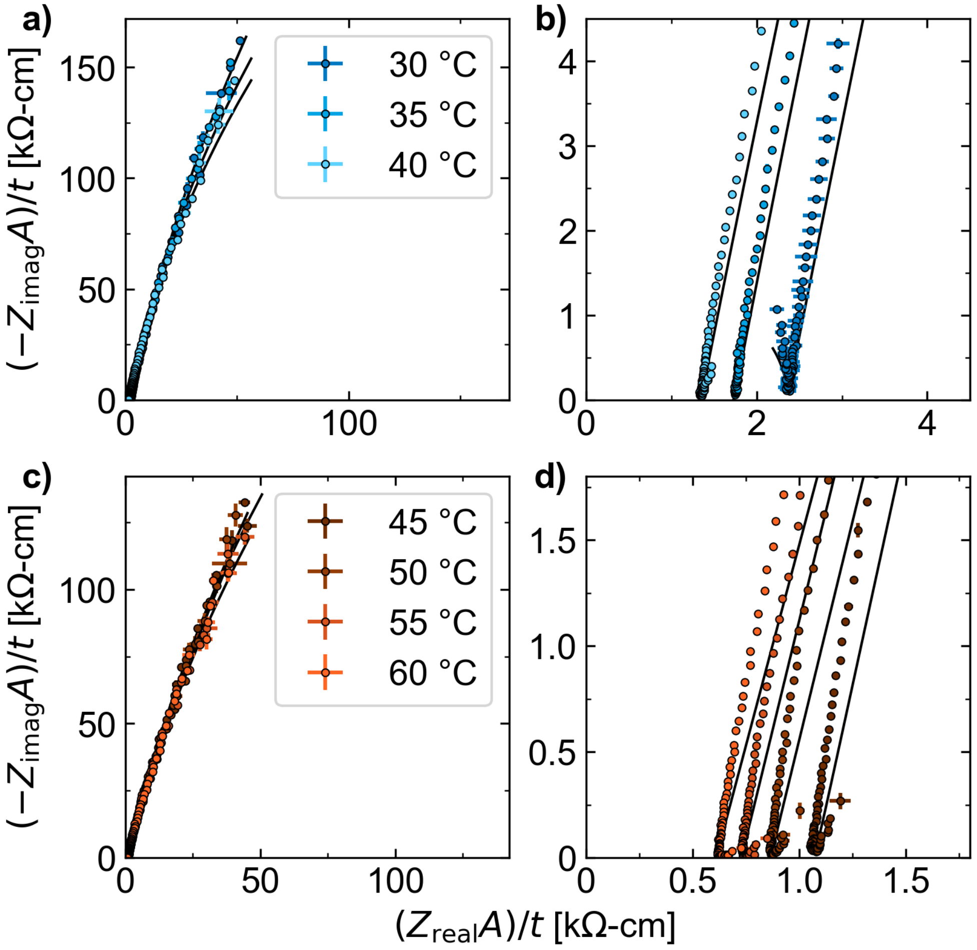

Figure S17: Nyquist plots for $Li_{1.42}Mg_{0.14}Zr_{0.57}Cl_4$ as a function of temperature, fit with an $R_1Q_1 + R_2Q_2$ equivalent circuit and normalized by sample area and thickness.

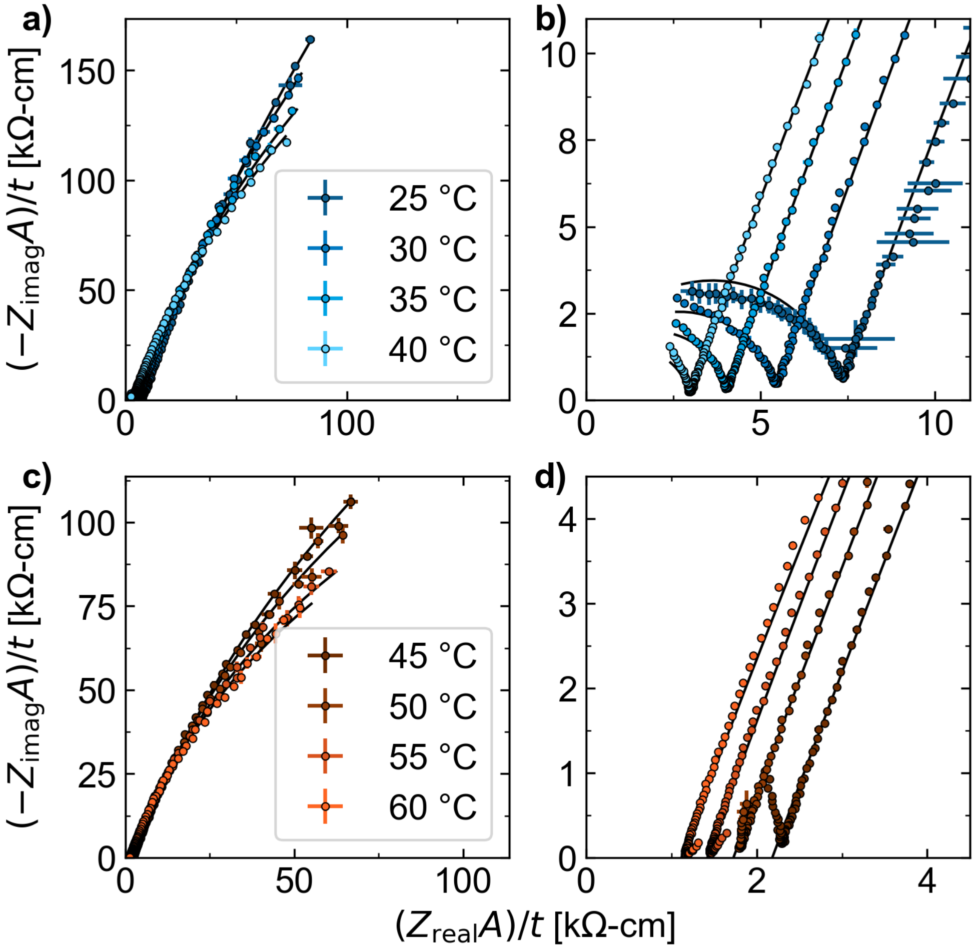

Figure S18: Nyquist plots for Li_2ZrCl_6 as a function of temperature, fit with an $R_1Q_1 + R_2Q_2$ equivalent circuit and normalized by sample area and thickness.

Some EIS measurements show unusual behavior in the high-frequency (ca. 10^6 Hz) region of the Nyquist plots when the $Z_{\mathrm{real}} < 2$ kΩ-cm, which we attribute to electronic interference from the surrounding environment. Specifically, the high-frequency semicircle of some measurements invert, meaning that it appears to the right of the x-intercept (see the 60 °C measurements in Figures S16-S18); normally, this tail appears to the left of the lowest point in $-Z_{\mathrm{imag}}$ (e.g., the 25 °C measurement in Figure S16). The electronic environment around the sample influences the high-frequency region of the EIS measurements. Comparing a $Li_{1.42}Mg_{0.14}Zr_{0.57}Cl_4$ ($z = 0.57$) sample measured inside the glovebox with the same sample measured outside the glovebox shows a substantial difference in the high-frequency region (Figure S19). Some of the shift can be attributed to a temperature change for "room temperature" inside the glovebox (28 °C) compared to outside (23 °C). However, the high frequency region changes more substantially than would be expected for temperature alone (See Figure S17). The tail for the measurement outside the glovebox points left, while the inside the glovebox measurement points to the right. As these measurements are nearly identical except for their surroundings, we attribute this difference in the Nyquist plot to the surroundings. As all the other EIS measurements we present in this work (Figures 5-7, S12-S18) were assessed in the same environment (an oven), any error caused by this noise is systematic.

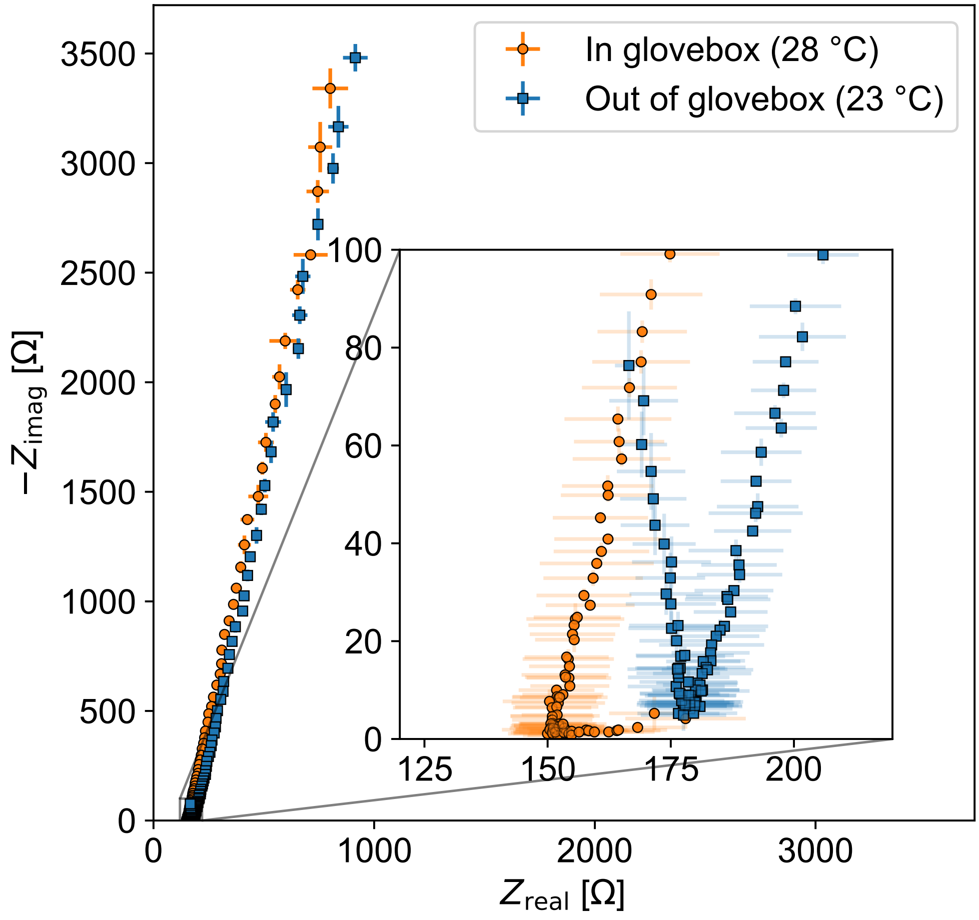

Figure S19: EIS measurements on $Li_{1.42}Mg_{0.14}Zr_{0.57}Cl_4$ ($x = 0.86$) inside the glovebox (orange circles) compared to outside the glovebox (blue squares). Measurements were conducted at room temperature, although that temperature was likely slightly higher inside the glovebox compared to outside the glovebox. Sample measured with steel contacts at 340 MPa.

BVSE calculations

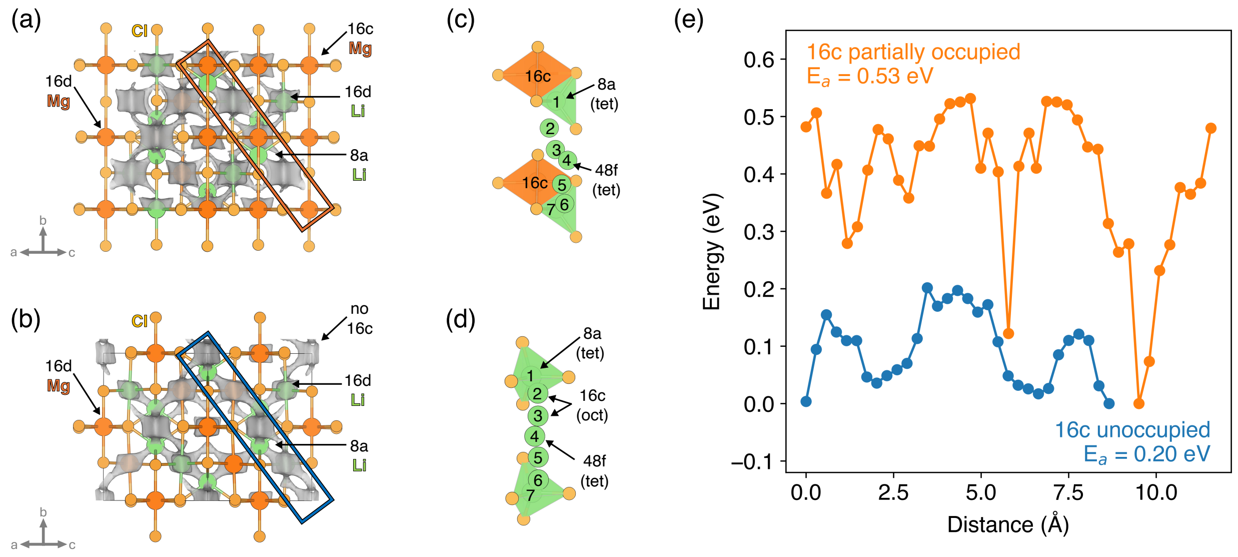

Figure S20: BVSE isosurfaces in (a) Li_2MgCl_4 spinel with partial Mg occupancy on 16*c* site, and (b) Li_2MgCl_4 spinel with no 16*c* occupancy. Li^+ 8*a* to 8*a* migration trajectories (c) and (d) without Mg^{2+} occupying the 16*c* site. (e) A comparison of migration energies between the two different paths.

To understand how cation occupancy in the 16*c* octahedral site affects ion transport in these spinels, we used bond valence site energy (BVSE) analysis to calculate lithium trajectories and migration energies. We chose Li_2MgCl_4 as a model system, as this phase is simpler to simulate than the quaternary materials and experimentally exhibited variable 16*c* site occupancy (Figure S6). To approximate the local environments of the disordered spinel structures, structural models with full site occupancy were generated using an open-source code.[14] Structural conformations with the lowest Coloumbic energy for both spinel types were selected for BVSE analysis (Figure S20a Mg^{2+} occupancy on both the 16*d* and 16*c* sites v. Figure S20b Mg^{2+} occupancy on the 16*d* site only). Isosurfaces and Li^+ migration trajectories were calculated using open source python software (BVlain).[15,16] As seen in Figure S20b, isosurface levels extend into the 16*c* site, whereas in Figure S20a they do not. This suggests that cation occupancy on the 16*c* site does in fact hinder the movement of Li ions and contribute to a higher migration energy. This can be more easily visualized through lithium trajectories of an 8*a* to 8*a* tetrahedral pathway (Figure S20c,d,e). If

the 16*c* site is occupied by Mg^{2+}, then coulombic repulsion results in a higher-energy pathway for migrating Li^+ ions (Figure S20c,e). In contrast, when the 16*c* site is unoccupied, Li^+ can easily move from one 8*a* site through the 16*c* and 48*f* interstitial voids to the next 8*a* site (Figure S20d,e). These calculations support the hypothesis that site disorder in the ball-milled spinels may inhibit lithium ion conductivity.

References

(1) Chupas, P. J.; Chapman, K. W.; Kurtz, C.; Hanson, J. C.; Lee, P. L.; Grey, C. P. A versatile sample-environment cell for non-ambient X-ray scattering experiments. *J. Appl. Crystallogr.* **2008**, *41*, 822–824.

(2) Schlem, R.; Muy, S.; Prinz, N.; Banik, A.; Shao-Horn, Y.; Zobel, M.; Zeier, W. G. Mechanochemical Synthesis: a Tool to Tune Cation Site Disorder and Ionic Transport Properties of Li_3MCl_6 (M= Y, Er) Superionic Conductors. *Adv. Energ. Mater.* **2020**, *10*, 1903719.

(3) Partik, M.; Schneider, M.; Lutz, H. Kristallstrukturen von $MgCr_2O_4$-Typ Li_2VCl_4 und Spinell-Typ Li_2MgCl_4 und Li_2CdCl_4. *Z. anorg. allg. Chem.* **1994**, *620*, 791–795.

(4) Banerjee, A.; Gupta, R.; Balani, K. Non-Monotonic Lattice Parameter Variation in Ball-Milled Ceria. *J. Mater. Sci.* **2015**, *50*, 6349–6358.

(5) Ott, H. Die Raumgitter der Lithiumhalogenide. *Phys. Z.* **1923**, *24*, 209–213.

(6) Partin, D.; O'Keeffe, M. The Structures and Crystal Chemistry of Magnesium Chloride and Cadmium chloride. *J. Solid State Chem.* **1991**, *95*, 176–183.

(7) Borjas Nevarez, R.; Balasekaran, S. M.; Kim, E.; Weck, P.; Poineau, F. Zirconium Tetrachloride Revisited. *Acta Crystallogr. C* **2018**, *74*, 307–311.

(8) Zhou, L.; Kwok, C. Y.; Shyamsunder, A.; Zhang, Q.; Wu, X.; Nazar, L. F. A New Halospinel Superionic Conductor for High-Voltage All Solid State Lithium Batteries. *Energ. Environ. Sci.* **2020**, *13*, 2056–2063.

(9) Yu, S.; Noh, J.; Kim, B.; Song, J.-H.; Oh, K.; Yoo, J.; Lee, S.; Park, S.-O.; Kim, W.; Kang, B., et al. Design of a Trigonal Halide Superionic Conductor by Regulating Cation Order-Disorder. *Science* **2023**, *382*, 573–579.

(10) Kwak, H.; Han, D.; Lyoo, J.; Park, J.; Jung, S. H.; Han, Y.; Kwon, G.; Kim, H.; Hong, S.-T.; Nam, K.-W., et al. New Cost-Effective Halide Solid Electrolytes for All-Solid-State Batteries: Mechanochemically Prepared Fe^{3+}-Substituted Li_2ZrCl_6. *Adv. Energ. Mater.* **2021**, *11*, 2003190.

(11) Luo, X.; Zhong, Y.; Wang, X.; Xia, X.; Gu, C.; Tu, J. Ionic Conductivity Enhancement of Li_2ZrCl_6 Halide Electrolytes via Mechanochemical Synthesis for All-Solid-State Lithium–Metal Batteries. *ACS Appl. Mater. Interfaces* **2022**, *14*, 49839–49846.

(12) Madsen, I. C.; Scarlett, N. V.; Cranswick, L. M.; Lwin, T. Outcomes of the International Union of Crystallography Commission on powder diffraction round robin on quantitative phase analysis: samples 1a to 1h. *Journal of Applied Crystallography* **2001**, *34*, 409–426.

(13) Kakaei, M. N.; Neshati, J.; Rezaierod, A. R. On the extraction of the effective capacitance from constant phase element parameters. *Protection of Metals and Physical Chemistry of Surfaces* **2018**, *54*, 548–556.

(14) Okhotnikov, K.; Charpentier, T.; Cadars, S. Supercell Program: a Combinatorial Structure-Generation Approach for the Local-Level Modeling of Atomic Substitutions and Partial Occupancies in Crystals. *Journal of cheminformatics* **2016**, *8*, 1–15.

(15) Dembitskiy, A. bvlain: The Bond Valence Site Energy Calculator. `https://github.com/dembart/BVlain`.

(16) Adams, S.; Rao, R. P. High Power Lithium Ion Battery Materials by Computational Design. *physica status solidi (a)* **2011**, *208*, 1746–1753.
